# Beyond Intentions: The Breastfeeding Intention-Practice Gap in Tunisia- A Cross-Sectional Analysis of Initiation and Continuation among Post-Partum Women

**DOI:** 10.12688/f1000research.169267.1

**Published:** 2025-09-09

**Authors:** Narjes karmous, Omar Oualha, Anouar Drira, Abdelwaheb Masmoudi, Badreddine Bouguerra, Abdennour Karmous

**Affiliations:** 1 Gynaecology and Obstetrics department B, Charles Niolle Hospital, Tunis, Tunisia; 2 Faculty of medicine of Tunis, University Tunis el Manar, Tunis, Tunisia; 3 Psychiatric department, Razi Hospital, Mannouba, Tunisia

**Keywords:** “Breastfeeding intention”, “Breastfeeding initiation”, “Breastfeeding continuation”

## Abstract

**Background:**

Breastfeeding is vital for maternal and child health, yet breastfeeding practices and duration vary globally. In Tunisia, data on factors influencing breastfeeding, especially initiation timing, are limited. This study assessed breastfeeding practices, initiation timing, and associated maternal and delivery factors among Tunisian women, focusing on breastfeeding duration up to 12 months postpartum.

**Methods:**

Analytical cross-sectional study was conducted over a four-month period, from November 1, 2023, to February 29, 2024, in the Gynecology and Obstetrics Department B of Charles Nicolle Hospital in Tunis- Tunisia. Women who delivered during the study period were included. Data on sociodemographic characteristics, medical and obstetric history, breastfeeding knowledge and preparation, delivery, postpartum and breastfeeding practices were collected through a questionnaire. Breastfeeding duration was grouped into 1–3, 3–6, and >6 months. Associations with breastfeeding duration were analyzed.

**Results:**

In total, 400 women were included. Most women were Tunisian (99%), urban residents (91.5%), and of higher socioeconomic status (84.3%). Obesity was present in 20.5%, and cesarean delivery rate was 52.8%. Early breastfeeding initiation (within 1 hour) occurred in only 19.3%, with 80.8% delayed initiation. Breastfeeding at 12 months was 44%. Early initiation was significantly associated with longer breastfeeding duration (p < 0.001); 77.9% of women initiating within the first hour continued breastfeeding at 12 months, compared to much lower rates with delayed initiation. Obesity predicted shorter breastfeeding duration (p = 0.026). cesarean delivery showed no significant impact. Skin-to-skin contact was low (38.3%), and less than half received breastfeeding education (46%). Family support was not linked to breastfeeding duration.

**Conclusions:**

Early breastfeeding initiation strongly supports sustained breastfeeding in Tunisia. Despite high breastfeeding intention, delays in initiation and modifiable barriers such as obesity and limited breastfeeding education hinder optimal breastfeeding outcomes. Interventions promoting early

## 1. Introduction

In 2025, breastfeeding remains one of the most powerful, yet underutilized interventions for improving global maternal and child health. Despite overwhelming evidence of its benefits, exclusive breastfeeding rates continue to fall short of targets, with only 48% of infants worldwide being exclusively breastfed for the recommended six months. ^1^ The gap between scientific evidence and real-world practice persists due to systemic barriers including inadequate healthcare support, workplace challenges, and persistent societal misconceptions.

Recent studies from 2023-2025 have further strengthened the case for breastfeeding, demonstrating its protective effects against childhood leukemia, ^2, 3^ food allergies, ^4^ and maternal diabetes. ^1^ However, in Tunisia, while breastfeeding initiation rates remain strong at 85%, exclusive breastfeeding at six months has plateaued at just 38%, reflecting regional trends and underscoring the need for targeted interventions. ^5^

The post-pandemic era has introduced new challenges, including healthcare workforce shortages and increased economic pressures that force mothers to return to work prematurely. At the same time, digital misinformation about infant feeding continues to proliferate, further complicating breastfeeding promotion efforts.

This study evaluated breastfeeding Tunisian practices in 2025, focusing on factors influencing breastfeeding continuation, particularly the timing of initiation. It addresses persistent barriers and highlights promising solutions, including Tunisia’s updated Labor Code for workplace lactation support and AI-powered lactation assistants. By reviewing successful interventions in similar contexts, this work offers actionable recommendations to close the implementation gap and accelerate progress toward global breastfeeding targets, vital for maternal and child health.

## 2. Methods

### 2.1 Study design and setting

Analytical cross-sectional study was conducted over a four-month period, from November 1, 2023, to February 29, 2024, in the Gynecology and Obstetrics Department B of Charles Nicolle Hospital in Tunis- Tunisia.

### 2.2 Study population

The study included women who delivered during the study period meeting the following criteria:

**Inclusion criteria**

• Age 18 years or older
• Delivery at 34 weeks of gestation or later, regardless of delivery mode
• Informed and voluntary consent to participate

**Exclusion criteria**

• Maternal age < 18 years
• Newborns transferred to specialized units (e.g., pediatric surgery or pediatric cardiology)
• Complicated deliveries requiring maternal admission to intensive care unit
• Intrauterine fetal death
• Neonatal death
• Medical contraindications to breastfeeding
• Refusal to participate
• Incomplete questionnaire data

**Figure 1.**
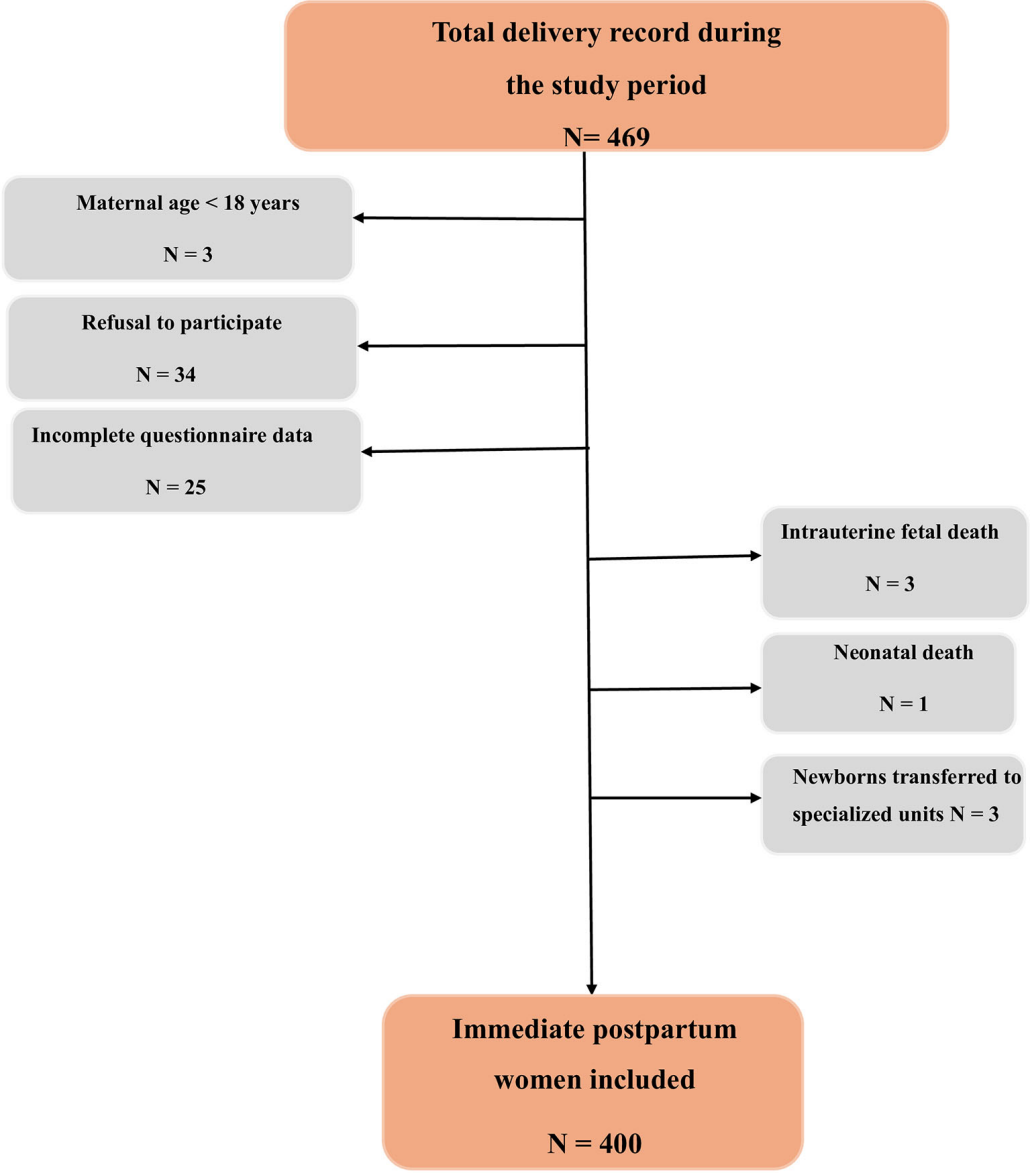
Flowchart for the study.

A study flowchart detailing case selection and exclusions has been developed ( Figure 1).

### 2.3 Variables

Data were collected through a structured, anonymous, self-administered questionnaire, conducted in the participant’s native language with informed consent and confidentiality strictly respected.

The questionnaire was organized into the following sections:

1. **Sociodemographic Characteristics:** Age, Ethnicity, Marital status, Place of residence, Education level, Socioeconomic status, Occupation
2. **Medical and Obstetric History:** Smoking, Body mass index (BMI), Medical history, Surgical history, Gravidity, Parity
3. **Breastfeeding Knowledge and Preparation:** Previous breastfeeding experience, Experience duration, Breastfeeding intention, Breastfeeding education, Importance of keeping baby with mother, Knowledge about breastfeeding …
4. **Delivery and Newborn Characteristics:** Number of babies, Mode of delivery, Type of anesthesia, Degree of urgency, Gestational age at birth, Birth weight, Secondary weight, Apgar score, Neonatal unit admission …
5. **Postpartum and Breastfeeding Practices:** Postpartum pain, Skin-to-skin contact, Breastfeeding initiation, Use of formula milk, Breastfeeding education, Family support, Total breastfeeding duration, Reason for stopping breastfeeding, Exclusive breastfeeding, Duration of exclusive breastfeeding, Breastfeeding continuation at 3, 6, and 12 months …

The primary outcomes of this study were on breastfeeding continuation at 3, 6, and 12 months postpartum as reported by maternal recall at 12 months postpartum.

### 2.4 Statistical analysis

Data were analyzed using SPSS software (version 26.0, IBM Corp), with Excel used for data presentation and visualization ( https://www.office.com/?omkt=fr-FR ).

Descriptive statistics summarized qualitative variables as frequencies and percentages. Quantitative variables were described using means ± standard deviations (for normally distributed data) or medians with interquartile ranges (for non-normal data). Normality was assessed using skewness, kurtosis, and formal tests such as the Shapiro-Wilk test. ^6^

Analytical methods included the Chi-squared test or Fisher’s exact test for categorical variables, and the Student’s t-test or Mann-Whitney U test for continuous variables depending on distribution. Statistical significance was set at p ≤ 0.05. ^7^

## 3. Results

In total, 400 women were included.

### 3.1 Descriptive study

**3.1.1 Demographic characteristics**

Most of the participants were Tunisian (396 women, 99.0%), while 4 women (1.0%) were of Sub-Saharan African origin. Regarding residence, 366 women (91.5%) lived in urban areas and 34 (8.5%) in rural areas. A higher education level was reported by 139 women (34.8%), whereas 261 (65.3%) did not have higher education. A low socioeconomic status was observed in 63 women (15.8%), while 337 (84.3%) had a higher socioeconomic level ( Table 1).

**Table 1.** Sociodemographic characteristics of the study population.

Demographic characteristics	Category	N	%
**Ethnicity**	**Tunisian**	396	99.0
	**Sub-Saharan African**	4	1.0
**Residence**	**Rural**	34	8.5
	**Urban**	366	91.5
**Higher Education**	**No**	261	65.3
	**Yes**	139	34.8
**Low Socioeconomic Level**	**No**	337	84.3
	**Yes**	63	15.8

**3.1.2 Health and lifestyle factors**

Table 2 represents health and lifestyle factors among the study population.

**Table 2.** Health and lifestyle factors among the study population.

Health & Lifestyle factors	Category	N	%
**Active Smoking**	**No smoking**	394	98.5
	**Smoking**	6	1.5
**Passive Smoking**	**No**	291	72.8
	**Yes**	109	27.3
**Tobacco Exposure**	**No exposure**	296	74.0
	**Active/passive exposure**	104	26.0
**Obesity**	**No obesity**	318	79.5
	**Obesity**	82	20.5
**Surgical History**	**No**	394	98.5
	**Appendectomy**	4	1.0
	**Cholecystectomy**	2	0.5

Active smoking was reported by 6 women (1.5%), while 394 (98.5%) did not smoke. Passive smoking exposure was reported by 109 women (27.3%), and 291 (72.8%) had no exposure. Overall tobacco exposure, whether active or passive, was reported by 104 women (26.0%), while 296 (74.0%) had no exposure.

Obesity was present in 82 women (20.5%), while 318 (79.5%) were not obese.

Regarding surgical history, 6 women (1.5%) had undergone surgery, with 4 (1.0%) having had an appendectomy and 2 (0.5%) a cholecystectomy. The remaining 394 women (98.5%) had no surgical history.

**3.1.3 Pregnancy and delivery details**

Pregnancy and delivery details among the study population are summarized in Table 3.

**Table 3.** Pregnancy and delivery details among the study population.

Pregnancy & Delivery details	Category	N	%
**Parity**	**Low parity (Pauciparous)**	211	52.8
	**Multiparous**	189	47.3
**Singleton/Multiple**	**Singleton**	396	99.0
	**Multiple**	4	1.0
**Delivery Mode**	**Cesarean section**	211	52.8
	**Vaginal delivery**	189	47.3
**Encouraged to Move During Labor**	**No**	34	8.5
	**Yes**	366	91.5
**Degree of Urgency**	**Urgent**	108	51.2
	**Non-urgent ("Cold")**	103	48.8
**Delivery Term (Weeks)**	**<37**	34	8.5
	**37-39**	234	58.5
	**39-40**	82	20.5
	**≥40**	50	12.5
**Delivery Term**	**Preterm**	34	8.5
	**Term**	366	91.5
**Macrosomia**	**No**	370	92.5
	**Yes**	30	7.5

Low parity (pauciparous) was reported in 211 women (52.8%), while 189 (47.3%) were multiparous.

A singleton pregnancy was reported in 396 women (99.0%) and a multiple pregnancy in 4 women (1.0%).

Cesarean section was the mode of delivery for 211 women (52.8%), while 189 (47.3%) had a vaginal delivery (AVB). During labor, 366 women (91.5%) were encouraged to move, whereas 34 (8.5%) were not.

Delivery was urgent in 108 cases (51.2%) and non-urgent (“cold”) in 103 cases (48.8%).

Regarding gestational age at delivery, 34 women (8.5%) gave birth before 37 weeks, 234 (58.5%) between 37 and 39 weeks, 82 (20.5%) between 39 and 40 weeks, and 50 (12.5%) at 40 weeks or more. In total, 34 deliveries (8.5%) were preterm, and 366 (91.5%) were term.

Macrosomia was present in 30 newborns (7.5%), while 370 (92.5%) did not have macrosomia.

**3.1.4 Postpartum and neonatal outcomes**

Table 4 represents postpartum and neonatal outcomes among the study population.

**Table 4.** Postpartum and neonatal outcomes details among the study population.

Postpartum & Neonatal outcomes	Category	N	%
**Neonatal Unit Admission**	**No**	320	80.0
	**Yes**	80	20.0
**Severe Postpartum Pain**	**No**	214	53.5
	**Yes**	186	46.5
**Skin-to-Skin Contact**	**No**	247	61.8
	**Yes**	153	38.3
**Mother-Child Separation**	**No**	327	81.8
	**Yes**	73	18.3

Admission to a neonatal unit was necessary for 80 newborns (20.0%), while 320 (80.0%) did not require it.

Severe postpartum pain was reported by 186 women (46.5%), while 214 (53.5%) did not report such pain.

Skin-to-skin contact was practiced by 153 women (38.3%), while 247 (61.8%) did not have this contact.

Mother-child separation occurred in 73 cases (18.3%), while 327 women (81.8%) remained with their newborns.

**3.1.5 Breastfeeding practices and support**

Breastfeeding practices and support among the study population are summarized in Table 5:

▪ **Breastfeeding experience** was reported by 176 women (44.0%), while 224 (56.0%) did not breastfeed.
▪ **Breastfeeding intention** was reported by 398 women (99.5%), and only 2 (0.5%) had no such intention.
▪ **Breastfeeding education** was received by 184 women (46.0%), while 216 (54.0%) did not receive any.
▪ **The timing of breastfeeding initiation** was as follows:
  - less than 1 hour for 77 women (19.3%),
  - between 1 and 6 hours for 210 (52.5%),
  - between 6 and 12 hours for 66 (16.5%),
  - between 12 and 24 hours for 5 (1.3%),
  - and 24 hours or more for 30 women (7.5%).
  - Twelve women (3.0%) did not initiate breastfeeding at all during hospitalisation.

**Table 5.** Breastfeeding practices and support among the study population.

Breastfeeding practices & Support	Category	N	%
**Breastfeeding Experience**	**No**	224	56.0
	**Yes**	176	44.0
**Intention to Breastfeed**	**No**	2	0.5
	**Yes**	398	99.5
**Breastfeeding Education**	**No**	216	54.0
	**Yes**	184	46.0
**Breastfeeding Initiation Delay**	**<1 hour**	77	19.3
	**1-6 hours**	210	52.5
	**6-12 hours**	66	16.5
	**12-24 hours**	5	1.3
	**≥24 hours**	30	7.5
	**No initiation during hospitalisation**	12	3.0
**Breastfeeding Initiation**	**Delayed**	323	80.8
	**Timely**	77	19.3
**Use of Formula Milk**	**No**	295	73.8
	**Yes**	105	26.3
**Assistance with First Feeding**	**No**	263	65.8
	**Yes**	137	34.3
**Assistance After First Feeding**	**No**	293	73.3
	**Yes**	107	26.8
**Education During Hospitalization**	**No**	293	73.3
	**Yes**	107	26.8
**Family Support**	**No**	110	27.5
	**Yes**	290	72.5

Overall, delayed initiation of breastfeeding was reported in 323 cases (80.8%), while timely initiation was noted in 77 cases (19.3%).

▪ **The use of formula milk** was reported by 105 women (26.3%), while 295 (73.8%) did not use it.
▪ **Assistance with the first feeding** was received by 137 women (34.3%), while 263 (65.8%) did not receive help. After the first feeding, further assistance was reported by 107 women (26.8%), while 293 (73.3%) did not receive additional help.
▪ **Breastfeeding education during hospitalization**: 107 women (26.8%) received breastfeeding education, while 293 (73.3%) did not.
▪ **Family support** was reported by 290 women (72.5%), while 110 (27.5%) did not receive support.

### 3.2 Analytical study

**3.2.1 Demographic and socioeconomic factors**

Table 6 represents the univariate analysis of demographic and socioeconomic factors across postpartum breastfeeding duration groups (1–3 months, 3–6 months, and >6 months).

**Table 6.** Univariate analysis of demographic and socioeconomic factors across postpartum breastfeeding duration groups (1–3 months, 3–6 months, and >6 months).

Demographic & Socioeconomic factors	Category	1–3 months N (%)	3–6 months N (%)	>6 months N (%)	p-value
**Ethnicity**	**Tunisian**	53 (100%)	104 (96.3%)	239 (100%)	0.064
	**Sub-Saharan African**	0 (0%)	4 (3.7%)	0 (0%)	
**Residence**	**Rural**	4 (7.5%)	11 (10.2%)	19 (7.9%)	0.740
	**Urban**	49 (92.5%)	97 (89.8%)	220 (92.1%)	
**Higher Education**	**No**	38 (71.7%)	69 (63.9%)	154 (64.4%)	0.525
	**Yes**	15 (28.3%)	39 (36.1%)	85 (35.6%)	
**Low Socioeconomic Level**	**No**	44 (83.0%)	80 (74.1%)	213 (89.1%)	**0.005**
	**Yes**	9 (17.0%)	28 (25.9%)	26 (10.9%)	

All participants in the 1–3 month and >6 month postpartum groups were Tunisian (100%), whereas in the 3–6 month group, 104 women (96.3%) were Tunisian and 4 women (3.7%) were Sub-Saharan African. This difference in ethnicity was not statistically significant ( *p* = 0.064).

Concerning residence, most women lived in urban areas across all groups: 92.5% in the 1–3 month group, 89.8% in the 3–6 month group, and 92.1% in the >6 month group. Rural residence was reported by 7.5%, 10.2%, and 7.9% of women respectively. These differences were not significant ( *p* = 0.740).

Regarding education, women without higher education represented 71.7% in the 1–3 month group, 63.9% in the 3–6 month group, and 64.4% in the >6 month group. These variations were not statistically significant ( *p* = 0.525).

However, socioeconomic status showed a significant difference between the groups ( *p* = 0.005). A low socioeconomic level was reported in 17.0% of women in the 1–3 month group, 25.9% in the 3–6 month group, and only 10.9% in the >6 month group.

**3.2.2 Health and lifestyle factors**

Table 7 represents the univariate analysis of healthand lifestyle factors across the postpartum breastfeeding duration groups.

**Table 7.** Univariate analysis of health and lifestyle factors across the postpartum breastfeeding duration groups (1–3 months, 3–6 months, and >6 months).

Health & Lifestyle factors	Category	1–3 months	3–6 months	>6 months	p-value
Active Smoking	No	51 (96.2%)	107 (99.1%)	236 (98.7%)	0.420
	Yes	2 (3.8%)	1 (0.9%)	3 (1.3%)	
Passive Smoking	No	35 (66.0%)	78 (72.2%)	178 (74.5%)	0.266
	Yes	18 (34.0%)	30 (27.8%)	61 (25.5%)	
Obesity	No	39 (73.6%)	80 (74.1%)	199 (83.3%)	**0.026**
	Yes	14 (26.4%)	28 (25.9%)	40 (16.7%)	

Active smoking was uncommon across all groups, with 3.8% of women smoking in the 1–3 month group, 0.9% in the 3–6 month group, and 1.3% in the >6 month group ( *p* = 0.420; not significant). Passive smoking exposure was reported by 34.0%, 27.8%, and 25.5% of women respectively ( *p* = 0.266; not significant).

Obesity showed a significant difference between groups ( *p* = 0.026). It was reported in 26.4% of women in the 1–3 month group, 25.9% in the 3–6 month group, and only 16.7% in the >6 month group.

**3.2.3 Pregnancy and delivery factors**

Cesarean section was the most common delivery mode in all groups, reported in 54.7% of the 1–3 month group, 52.8% of the 3–6 month group, and 52.3% of the >6 month group ( *p* = 0.789; not significant).

Preterm delivery (<37 weeks) occurred in 9.4%, 9.3%, and 7.9% of the women, respectively ( *p* = 0.635; not significant).

Macrosomia was similarly distributed among groups (5.7%, 8.3%, and 7.5%; *p* = 0.864; not significant) (See Table 8).

**Table 8.** Univariate analysis of pregnancy and delivery factors across postpartum breastfeeding duration groups (1–3 months, 3–6 months, and >6 months).

Pregnancy & Delivery factors	Category	1–3 months	3–6 months	>6 months	p-value
Delivery Mode	**Cesarean section**	29 (54.7%)	57 (52.8%)	125 (52.3%)	0.789
	**Vaginal delivery**	24 (45.3%)	51 (47.2%)	114 (47.7%)	
Term Delivery	**Preterm (<37 weeks)**	5 (9.4%)	10 (9.3%)	19 (7.9%)	0.635
	**Term (≥37 weeks)**	48 (90.6%)	98 (90.7%)	220 (92.1%)	
Macrosomia	**No**	50 (94.3%)	99 (91.7%)	221 (92.5%)	0.864
	**Yes**	3 (5.7%)	9 (8.3%)	18 (7.5%)	

**3.2.4 Breastfeeding practices and support**

The univariate analysis of breastfeeding practices and support factors across postpartum breastfeeding duration groups (1–3 months, 3–6 months, and >6 months) is summarized in Table 9:

▪ **Breastfeeding initiation:**
  - **Timely breastfeeding initiation (within 1 hour)** showed a significant difference between groups ( *p* < 0.001). Only 13.2% of women in the 1–3-month group and 2.8% in the 3–6 month group initiated breastfeeding early, compared to 28.0% in the >6-month group.
  - Consequently, **delayed breastfeeding initiation** was more prevalent in the 1–3 month (86.8%) and 3–6-month (97.2%) groups than in the >6-month group (72.0%), which was also statistically significant ( *p* < 0.001).
▪ **The use of formula milk** was reported by 34.0% of women in the 1–3-month group, 22.2% in the 3–6 month group, and 26.4% in the >6-month group ( *p* = 0.750; not significant).
▪ **Skin-to-skin contact** was practiced in 37.7%, 36.1%, and 39.3% of the cases in the respective groups ( *p* = 0.633; not significant).
▪ **Family support** was present for 79.2% of the 1–3 month group, 71.3% of the 3–6 month group, and 71.5% of the >6-month group ( *p* = 0.450; not significant).
▪ **Breastfeeding continuation**

**Table 9.** Univariate analysis of breastfeeding practices and support factors across postpartum breastfeeding duration groups (1–3 months, 3–6 months, and >6 months).

Breastfeeding practices & Support	Category	1–3 months	3–6 months	>6 months	p-value
Timely Initiation (<1h)	Yes	7 (13.2%)	3 (2.8%)	67 (28.0%)	**0.000**
Delayed Initiation	Yes	46 (86.8%)	105 (97.2%)	172 (72.0%)	**0.000**
Formula Use	Yes	18 (34.0%)	24 (22.2%)	63 (26.4%)	0.750
Skin-to-Skin Contact	Yes	20 (37.7%)	39 (36.1%)	94 (39.3%)	0.633
Family Support	Yes	42 (79.2%)	77 (71.3%)	171 (71.5%)	0.450

**Figure 2.**
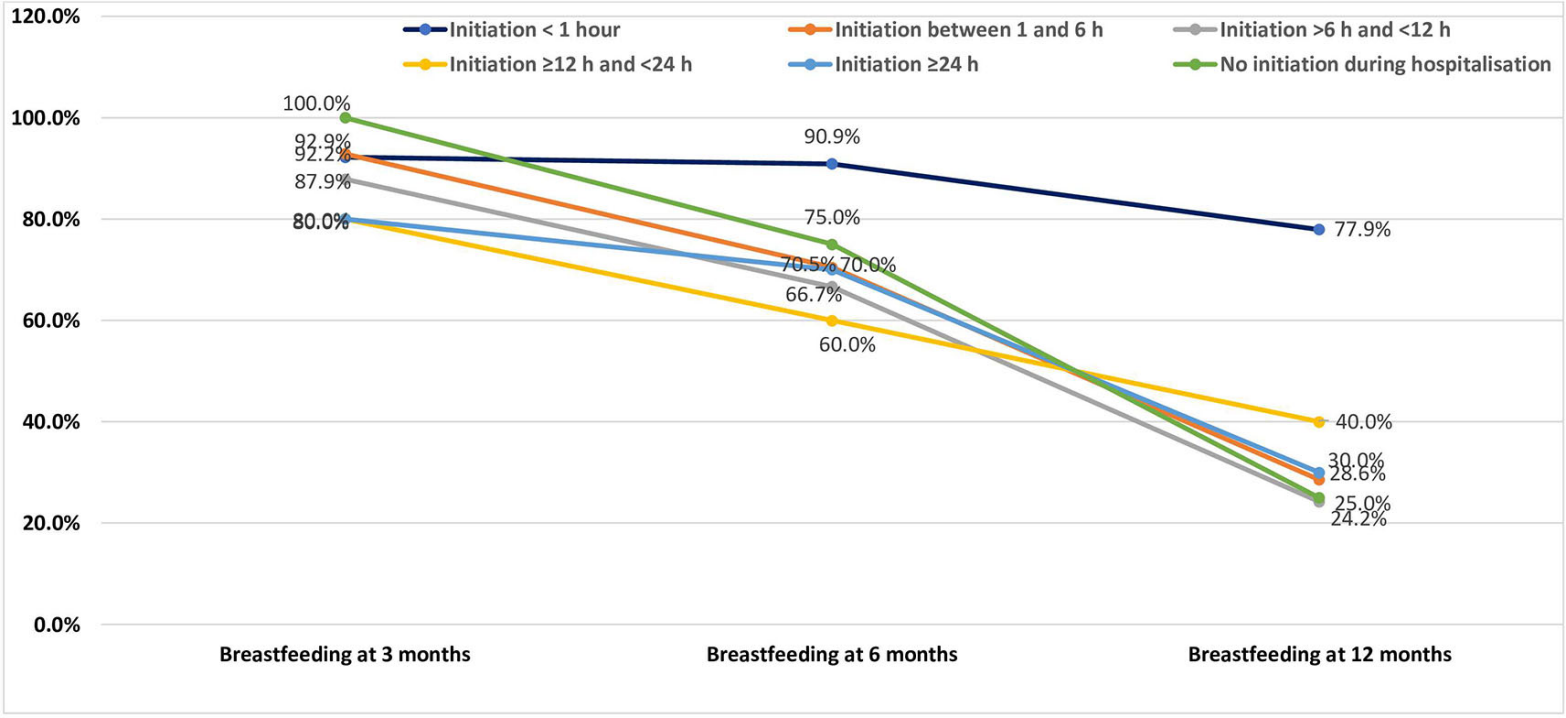
Breastfeeding duration in relation to the timing of breastfeeding initiation among the study population.

An analysis of breastfeeding duration in relation to the timing of breastfeeding initiation reveals a clear trend: the earlier the initiation, the higher the likelihood of sustained breastfeeding, particularly at 6 and 12 months ( Figure 2).

At 3 months postpartum, breastfeeding rates remained relatively high across all initiation groups. Women who initiated breastfeeding within the first hour had a breastfeeding rate of 92.2%, comparable to those who initiated between 1 and 6 hours (92.9%). Even among those who initiated breastfeeding after 24 hours or did not initiate at all during hospitalisation, rates remained high (80.0% and 100.0%, respectively). This suggests that in the short term, other factors—such as initial motivation or support in the early postpartum period—may help maintain breastfeeding regardless of initiation timing.

However, differences became more pronounced at 6 months. Women who initiated breastfeeding within the first hour had the highest rate (90.9%), whereas rates dropped to 70.5% among those who initiated between 1 and 6 hours, and to 66.7% for those initiating between 6 and 12 hours. The lowest rates were seen among those with later initiation (60–70%) and no initiation (75%). These findings suggest that early initiation supports better breastfeeding continuity.

At 12 months, the trend was even more marked. The breastfeeding rate remained relatively high in the group that initiated within the first hour (77.9%), while it fell drastically in all other groups. Only 28.6% of women who initiated between 1 and 6 hours, 24.2% of those between 6 and 12 hours, and 25.0% of those who did not initiate at all during hospitalisation were still breastfeeding at 12 months. This demonstrates the long-term benefit of early breastfeeding initiation, especially within the first hour of birth.

## 4. Discussion

The findings from our study examining breastfeeding practices among Tunisian women reveal important similarities and differences when compared with international research.

Our observation of a 44% breastfeeding rate at 12 months postpartum aligns closely with Yalçin et al.’s findings ^8^ in Turkey (55.9%), yet surpasses rates reported in high-income countries like Australia (31.8%) ^9^ and Korea (33.7%). ^10^ This suggests cultural factors in Tunisia may be more supportive of prolonged breastfeeding than in some Western contexts, though substantial room for improvement remains.

A critical finding from our study was the powerful association between early initiation and breastfeeding duration - while only 19.3% of mothers initiated breastfeeding within the first hour, this group accounted for 77.9% of those still breastfeeding at 12 months. This dose-response relationship echoes Meedya’s intervention research ^11^ showing early practices significantly influence long-term outcomes.

However, our 80.8% delayed initiation rate indicates systemic challenges in immediate postpartum care that mirror Bond et al.’s identification of hospital routines as barriers. ^12^

Our analysis of breastfeeding determinants revealed both expected and novel findings compared to existing literature.

The socioeconomic gradient we observed confirms Chimoriya et al.’s Sydney-based findings, ^13^ though the mechanisms may differ in our context.

Our study identified obesity (present in 20.5% of participants) as a significant predictor of shorter breastfeeding duration, consistent with the findings of Achike and Akpinar-Elci’s systematic review, ^14^ which analyzed 23 studies on the impact of pregestational maternal BMI on breastfeeding outcomes. Their review showed that elevated maternal BMI prior to pregnancy is linked to reduced intention to breastfeed, delayed initiation, and shorter duration of both exclusive and overall breastfeeding. This association can be attributed to a combination of physiological barriers (delayed lactogenesis, hormonal dysregulation), psychological challenges (low confidence, negative body image), and systemic factors (less support, provider bias). ^14^

Contrary to expectations from Bond et al.’s work, ^12^ we found no association between cesarean delivery (52.8% rate) and breastfeeding outcomes, suggesting cultural or healthcare adaptations may mitigate this typically negative relationship.

The postpartum practices documented in our study highlight several evidence-practice gaps. Our low skin-to-skin contact rate (38.3%) falls far below standards advocated by the American Academy of Pediatrics, ^15^ while limited breastfeeding education (46%) contrasts with Park and Ryu’s ^16^ meta-analysis showing education’s effectiveness.

These implementation shortcomings help explain our population’s challenges, particularly when combined with Niu et al.’s findings ^17^ about the importance of early metabolic programming through breastfeeding.

Our health outcome data contribute new perspectives to existing research. While Delgado et al. ^18^ focused on long-term mental health outcomes and Niu et al. ^17^ examined metabolic benefits, our documentation of immediate postpartum challenges (20% neonatal intensive care unit admission, 46.5% severe pain) identifies understudied barriers to breastfeeding establishment. These findings suggest Cordell and Elverson’s (2021) call for improved postpartum support applies particularly strongly in our context. ^19^

The policy implications emerging from our study both confirm and extend existing recommendations. Our results strongly support implementing the World Health Organisation (WHO)’s “Ten Steps” as analyzed by Binns and Lee, ^20^ while also suggesting needed adaptations for local factors like obesity prevalence.

The limited impact of family support in our study contrasts with Scott et al.’s ^9^ emphasis on partner involvement, indicating Tunisian programs may need different support strategies than Western models.

Our research makes several unique contributions to the literature. The detailed initiation timing analysis provides granularity beyond most cohort studies, while the Tunisian context offers an important addition to research dominated by Western and Asian populations.

Our unexpected findings regarding cesarean delivery and family support challenge some assumptions in the current literature, particularly the Section on Breastfeeding’s universal recommendations. ^21^ These results suggest future research should test culturally-adapted interventions while maintaining rigorous outcome measurement as advocated by Bonnet et al. ^22^

Ultimately, our study reinforces that effective breastfeeding support requires both evidence-based practices and cultural adaptation. While universal biological principles exist, as highlighted by Kramer et al.’s ^23^ growth studies, their implementation must account for local realities - from hospital routines to family dynamics. This balanced approach offers the best promise for improving breastfeeding outcomes in Tunisia and beyond.

### 4.1 Study strengths and limitations

Our study of breastfeeding practices among Tunisian women makes several important contributions to the literature while acknowledging certain limitations that contextualize the findings.

Among its principal strengths is the provision of rare data from a North African context, filling a geographical gap in breastfeeding research that has traditionally been dominated by studies from Western and Asian populations. ^24^

By documenting both the high breastfeeding intention (99.5%) and the substantial drop in actual practice (44% at 12 months), our work captures the challenging transition from desire to implementation that has been observed in diverse settings but rarely studied in our region.

The detailed categorization of breastfeeding initiation timing represents another significant strength, allowing us to demonstrate the dramatic impact of early initiation - with the 19.3% of mothers who began breastfeeding within the first hour showing a remarkable 77.9% continuation rate at 12 months. This finding provides strong local evidence supporting global recommendations about the “Golden Hour” while also revealing critical gaps in hospital practices, as 80.8% of mothers experienced delayed initiation.

The clinical relevance of our findings is enhanced by the identification of modifiable barriers, particularly the association between maternal obesity (present in 20.5% of our sample) and reduced breastfeeding duration - a relationship that has physiological plausibility but has been underemphasized in many population-level studies.

Similarly, our documentation of severe postpartum pain in 46.5% of mothers highlights an immediate challenge to breastfeeding establishment that has received limited attention in the intervention literature. These findings collectively offer actionable targets for quality improvement initiatives in our healthcare system.

From a policy perspective, our results both confirm the universal importance of early breastfeeding practices and challenge some assumptions about determinants like family support, suggesting the need for culturally adapted approaches that may differ from Western models.

However, several limitations must be acknowledged when interpreting these findings.

Our sample’s limited ethnic diversity (99% Tunisian) and urban predominance (91.5%) constrain the generalizability of results to more diverse or rural populations, where different barriers might prevail.

The reliance on maternal recall for breastfeeding duration rather than longitudinal tracking introduces potential reporting bias, though this is a common limitation shared with many similar studies.

Certain important confounders were not measured, including maternal employment status and specific hospital policies like Baby-Friendly designation, which have been shown to significantly influence breastfeeding outcomes in other settings.

The high cesarean rate in our sample (52.8%) may reflect local obstetric practices that differ from other regions, potentially limiting the generalizability of our finding that surgical delivery did not affect breastfeeding outcomes.

Finally, the cross-sectional design prevents definitive causal interpretations of the observed associations, while the lack of data on breastfeeding intensity (exclusive vs. partial) makes direct comparison with WHO guidelines more challenging.

Despite these limitations, the strengths of our culturally specific data and detailed analysis of initiation timing provide valuable insights that can inform both clinical practice and future research in our region. The unexpected patterns we observed regarding factors like family support and cesarean delivery challenge simplistic assumptions about universal determinants of breastfeeding success and highlight the importance of understanding local context.

While our findings support the implementation of certain evidence-based practices like immediate skin-to-skin contact, they also suggest the need for tailored interventions addressing unique local barriers such as high rates of postpartum pain and obesity.

Future research should build on these findings through longitudinal designs with more diverse samples and objective outcome measures, while our results can immediately inform efforts to improve breastfeeding support in Tunisian healthcare settings.

The study ultimately demonstrates how global breastfeeding recommendations must be adapted to local realities to achieve meaningful improvements in maternal and child health outcomes.

### 4.2 Implications

Our findings reveal a substantial gap between the high maternal intention to breastfeed and the actual breastfeeding practices observed in the first year postpartum. Clinically, this gap points to several modifiable factors that healthcare providers can address within routine obstetric and pediatric care.

1. **Early initiation of breastfeeding as a clinical priority:** The strong positive association between early initiation of breastfeeding—especially within the first hour of life—and sustained breastfeeding duration underscores the need for systematic implementation of *“Golden Hour”* practices. This includes ensuring immediate and uninterrupted skin-to-skin contact and avoiding non-medically justified mother–infant separation, even in cesarean deliveries when the mother’s condition allows.
2. **Integration of skin-to-skin contact into standard delivery room practice:** The relatively low rates of skin-to-skin contact and early initiation highlight a shortfall in adherence to WHO Baby-Friendly Hospital Initiative (BFHI) standards. ^25, 26^ Maternity units should prioritize staff training on evidence-based lactation support, establish regular performance audits, and integrate breastfeeding indicators into routine hospital quality metrics.
3. **Targeted support for at-risk populations:** Maternal obesity emerged as a risk factor for shorter breastfeeding duration, indicating the need for targeted antenatal counseling on breastfeeding readiness, positioning, and latch optimization. This can be achieved through multidisciplinary collaboration between obstetricians, midwives, lactation consultants, and nutritionists.
4. **Addressing postpartum pain management:** Nearly half of the mothers reported severe postpartum pain, which may impede early breastfeeding. Effective pain management protocols—using both pharmacologic and non-pharmacologic strategies—should be integrated into postnatal care without compromising infant safety.
5. **Workplace lactation support:** Beyond hospital discharge, workplace lactation support is a critical determinant of breastfeeding continuation for employed mothers. Recent amendments to Tunisia’s Labor Code create a policy opportunity for health professionals to advocate for and guide the implementation of employer-based lactation facilities, flexible breaks, and awareness campaigns that normalize breastfeeding in public and professional spaces.

### 4.3 Recommendations for further research

While our study provides important insights, several areas warrant additional investigation to inform policy and practice:

1. **Longitudinal cohort studies** following mother–infant pairs beyond 12 months to evaluate determinants of breastfeeding continuation into the second year, as recommended by WHO.
2. **Intervention trials** testing the effectiveness of structured hospital-based programs (e.g., immediate skin-to-skin, pain management, targeted lactation counseling) on improving breastfeeding initiation and duration.
3. **Qualitative studies** exploring sociocultural beliefs, family dynamics, and healthcare provider attitudes that influence breastfeeding practices in Tunisian and comparable North African contexts.
4. **Economic evaluations** to assess the cost-effectiveness of implementing BFHI standards and workplace lactation support measures at a national scale.
5. **Digital health innovations research** to examine the acceptability, accessibility, and impact of AI-assisted lactation support tools, particularly for working mothers and those with limited access to in-person consultation.

## 5. Conclusions

This study provides robust, locally relevant evidence that early initiation of breastfeeding within the first hour after birth plays a decisive role in sustaining breastfeeding through the first year of life in Tunisia.

Despite high maternal motivation, systemic barriers—including delayed initiation, low skin-to-skin contact, insufficient breastfeeding education, maternal obesity, and inadequate postpartum pain management—continue to undermine breastfeeding outcomes.

Strengthening early postpartum hospital practices, ensuring continuous professional support, and leveraging recent policy advances on workplace lactation are essential steps toward bridging the gap between breastfeeding intention and practice. Aligning Tunisian maternity care with international best practices could significantly improve maternal–child health and contribute to achieving global breastfeeding targets.

## Ethical considerations

Ethical approval for this study was obtained from the Institutional Ethics Committee of Charles Nicolle Hospital, Tunis, Tunisia (Approval number: FWA00032748 – IORG0011243) on October 24, 2023, prior to data collection.

## Consent to participate

Verbal informed consent was selected instead of written consent due to the specific context of the study population. Data were collected from postpartum women during the immediate or early postnatal period, when participants were often physically exhausted, emotionally vulnerable, and had limited time. Requesting written consent in this setting risked creating additional stress or discouraging participation, particularly among women with lower literacy levels.

This approach was reviewed and approved by the Institutional Ethics Committee of Charles Nicolle Hospital as the study involved minimal risk, collected no identifying or sensitive information, and ensured complete anonymity of responses.

All participants received clear information about the study objectives and procedures. Verbal informed consent was obtained, emphasizing the voluntary nature of participation and the right to withdraw at any time without consequence. Confidentiality was strictly maintained: no identifying information was collected, and all responses were recorded anonymously.

## Data Availability

All data sets can be assessed and all study findings reported in the article are shared via Harvard. Harvard Dataverse: “Beyond Intentions: The Breastfeeding Intention-Practice Gap in Tunisia- A Cross-Sectional Analysis of Initiation and Continuation among Post-Partum Women”, https://doi.org/10.7910/DVN/PXCDRZ. ^27^ This project contains the following:
• Dataset Breastfeeding final English • Breastfeeding findings Harvard Dataverse: “Beyond Intentions: The Breastfeeding Intention-Practice Gap in Tunisia- A Cross-Sectional Analysis of Initiation and Continuation among Post-Partum Women”, https://doi.org/10.7910/DVN/PXCDRZ. ^27^ This project contains the following:
• Breastfeeding Questionnaire. Data are available under the terms of the Creative Commons Zero “No rights reserved” data waiver (CC0 1.0 Public domain dedication).
