# Factors Associated with Breastfeeding Duration in Tunisia: A Cross-Sectional Analysis Highlighting the Role of Early Initiation

**DOI:** 10.12688/f1000research.169267.2

**Published:** 2025-12-12

**Authors:** Narjes karmous, Omar Oualha, Anouar Drira, Abdelwaheb Masmoudi, Badreddine Bouguerra, Abdennour Karmous

**Affiliations:** 1 Gynaecology and Obstetrics department B, Charles Niolle Hospital, Tunis, Tunisia; 2 Faculty of medicine of Tunis, University Tunis el Manar, Tunis, Tunisia; 3 Psychiatric department, Razi Hospital, Mannouba, Tunisia

**Keywords:** “Breastfeeding intention”, “Breastfeeding initiation”, “Breastfeeding continuation”

## Abstract

**Background:**

Breastfeeding is vital for maternal and child health, yet breastfeeding practices and duration vary globally. In Tunisia, data on factors influencing breastfeeding, especially initiation timing, are limited. This study assessed breastfeeding practices, initiation timing, and associated maternal and delivery factors among Tunisian women, focusing on breastfeeding duration up to 12 months postpartum.

**Methods:**

Analytical cross-sectional study was conducted over a four-month period, from November 1, 2023, to February 29, 2024, in the Gynecology and Obstetrics Department B of Charles Nicolle Hospital in Tunis- Tunisia. Women who delivered during the study period were included. Data on sociodemographic characteristics, medical and obstetric history, breastfeeding knowledge and preparation, delivery, postpartum and breastfeeding practices were collected through a questionnaire. Breastfeeding duration was grouped into 1–3, 3–6, and >6 months. Associations with breastfeeding duration were analyzed.

**Results:**

In total, 400 women were included. Most women were Tunisian (99%), urban residents (91.5%), and of higher socioeconomic status (84.3%). Obesity was present in 20.5%, and cesarean delivery rate was 52.8%. Early breastfeeding initiation (within 1 hour) occurred in only 19.3%, with 80.8% delayed initiation. Breastfeeding continuation at 12 months was 44% and was significantly associated with early initiation (p < 0.001); 77.9% of women who initiated breastfeeding within the first hour continued at 12 months, compared to markedly lower rates among those with delayed initiation. Maternal obesity predicted shorter breastfeeding duration (p = 0.026). Cesarean delivery showed no significant impact. Skin-to-skin contact was low (38.3%), and less than half received breastfeeding education (46%). Family support was not linked to breastfeeding duration.

**Conclusions:**

Breastfeeding continuation at 12 months was associated with early initiation and negatively influenced by maternal obesity. Despite high breastfeeding intention, delays in initiation, obesity and limited breastfeeding education hinder optimal breastfeeding outcomes. Interventions promoting early initiation, skin-to-skin contact, and tailored postnatal support are needed to improve breastfeeding continuation.

## 1. Introduction

In the last decade, breastfeeding remains one of the most powerful, yet underutilized interventions for improving global maternal and child health. Despite overwhelming evidence of its benefits, exclusive breastfeeding rates continue to fall short of targets, with only 48% of infants worldwide being exclusively breastfed for the recommended six months. ^1^ This gap between scientific evidence and real-world practice is even more pronounced in the Middle East and North Africa (MENA), where only ~35% of infants aged 0–5 months are exclusively breastfed according to regional UNICEF strategic data. ^2^ Systemic barriers including inadequate healthcare support, workplace challenges, and persistent societal misconceptions further contribute to low breastfeeding rates.

Recent studies from 2023-2025 have further strengthened the case for breastfeeding, demonstrating its protective effects against childhood leukemia, ^3, 4^ food allergies, ^5^ and maternal diabetes. ^1^ However, in Tunisia, while breastfeeding initiation rates remain strong at 85%, exclusive breastfeeding at six months has plateaued at just 38%, reflecting regional trends and underscoring the need for targeted interventions. ^6^

The post-pandemic era has introduced new challenges, including healthcare workforce shortages and increased economic pressures that force mothers to return to work prematurely. At the same time, digital misinformation about infant feeding continues to proliferate, further complicating breastfeeding promotion efforts.

Beyond structural and cultural barriers, behavioral determinants play a critical role in shaping breastfeeding outcomes. The observed discrepancy between a mother’s intention to breastfeed and her actual practice—the so-called *intention–practice gap*—can be theoretically understood through the Theory of Planned Behavior (TPB) and the Health Belief Model (HBM). According to Ajzen’s TPB, behavioral intention depends on attitudes, subjective norms, and perceived control, which may be constrained by social or institutional barriers. ^7^ Likewise, Rosenstock’s HBM emphasizes that perceived benefits, barriers, and cues to action influence whether health-promoting behaviors such as sustained breastfeeding are adopted. ^8^ Applying these models offers a conceptual framework for understanding why, despite strong maternal intentions, breastfeeding continuation remains suboptimal.

In addition to behavioral support, structural interventions are essential. Human milk banks—which provide screened donor milk for infants unable to receive their mother’s milk, especially preterm or low-birth-weight newborns—have been successfully implemented in several low- and middle-income countries. ^9, 10^ Although milk banking initiatives are still emerging in North Africa, integrating them into maternal and neonatal health systems could complement breastfeeding promotion and improve neonatal outcomes. ^11^

The study included women who delivered between November 2023 and February 2024. Breastfeeding continuation was assessed up to 12 months postpartum, with data collection extending into early 2025, focusing on factors influencing breastfeeding continuation, particularly the timing of initiation. It addresses persistent barriers and highlights promising solutions, including Tunisia’s updated Labor Code for workplace lactation support and AI-powered lactation assistants. By reviewing successful interventions in similar contexts, this work offers actionable recommendations to close the implementation gap and accelerate progress toward global breastfeeding targets, vital for maternal and child health.

## 2. Methods

### 2.1 Study design and setting

This analytical study was conducted at the Gynecology and Obstetrics Department B of Charles Nicolle Hospital, Tunis, Tunisia. The study period, defined as the period of participant inclusion, was from November 1, 2023, to February 29, 2024, during which all women delivering at the department were consecutively invited to participate. Baseline sociodemographic, obstetric, and early postpartum breastfeeding data were collected within the immediate postpartum period. Breastfeeding duration up to 12 months postpartum was obtained retrospectively by maternal recall at the 12-month mark. Therefore, the study represents a cross-sectional analysis with retrospective ascertainment of breastfeeding duration.

### 2.2 Study population

The study included women who delivered during the study period meeting the following criteria:

**Inclusion criteria**

• Age 18 years or older
• Delivery at 34 weeks of gestation or later, regardless of delivery mode
• Informed and voluntary consent to participate

**Exclusion criteria**

• Maternal age < 18 years
• Newborns transferred to specialized units (e.g., pediatric surgery or pediatric cardiology)
• Complicated deliveries requiring maternal admission to intensive care unit
• Intrauterine fetal death
• Neonatal death
• Medical contraindications to breastfeeding
• Refusal to participate
• Incomplete questionnaire data

**Figure 1.**
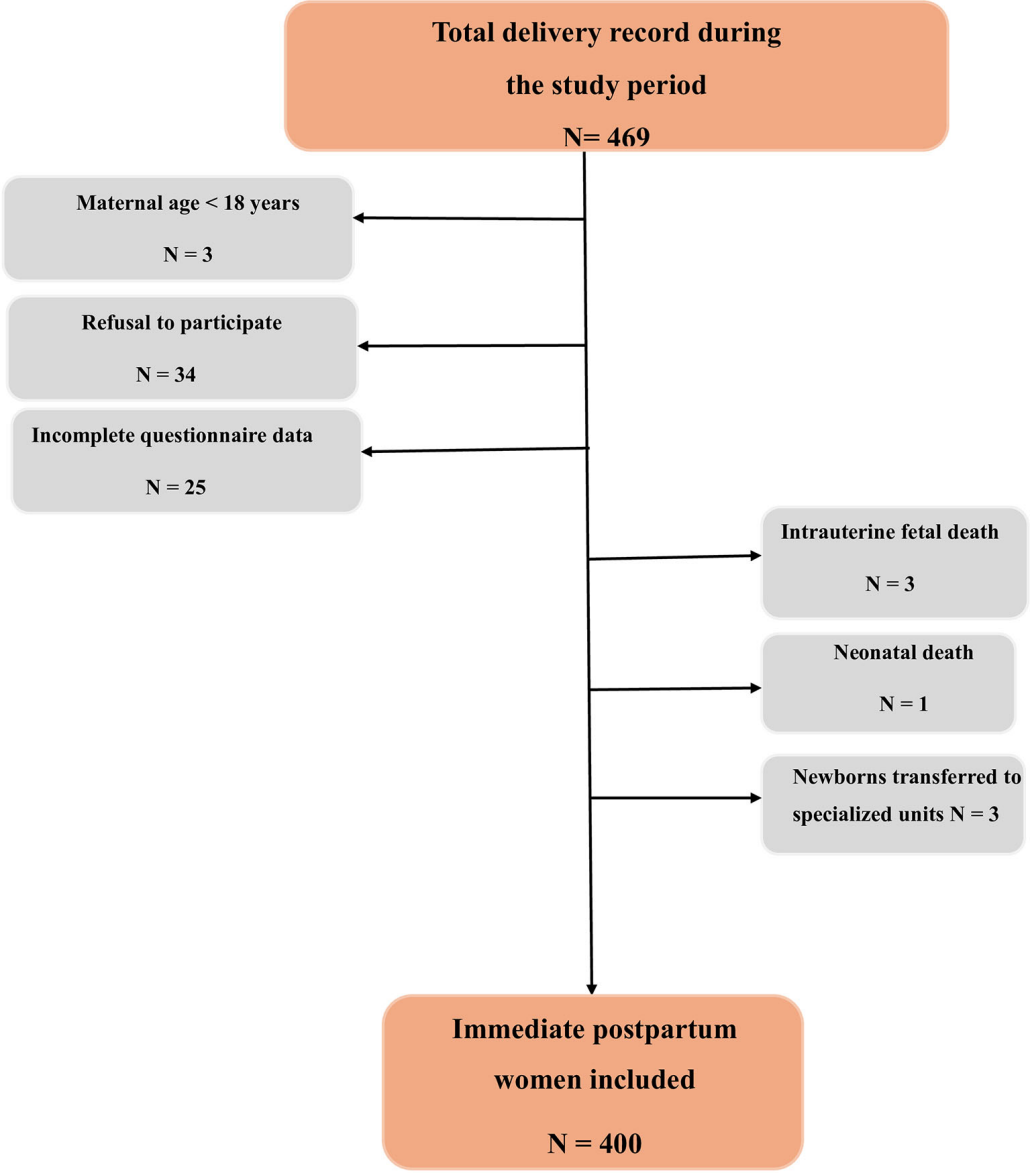
Flowchart for the study.

A study flowchart detailing case selection and exclusions has been developed ( Figure 1).

### 2.3 Variables

Data were collected through a structured, anonymous, self-administered questionnaire, conducted in the participant’s native language with informed consent and confidentiality strictly respected.

The questionnaire was organized into the following sections:

1. **Sociodemographic Characteristics:** Age, Ethnicity, Marital status, Place of residence, Education level, Socioeconomic status, Occupation
2. **Medical and Obstetric History:** Smoking, Body mass index (BMI), Medical history, Surgical history, Gravidity, Parity
3. **Breastfeeding Knowledge and Preparation:** Previous breastfeeding experience, Experience duration, Breastfeeding intention, Breastfeeding education, Importance of keeping baby with mother, Knowledge about breastfeeding …
4. **Delivery and Newborn Characteristics:** Number of babies, Mode of delivery, Type of anesthesia, Degree of urgency, Gestational age at birth, Birth weight, Secondary weight, Apgar score, Neonatal unit admission …
5. **Postpartum and Breastfeeding Practices:** Postpartum pain, Skin-to-skin contact, Breastfeeding initiation, Use of formula milk, Breastfeeding education, Family support, Total breastfeeding duration, Reason for stopping breastfeeding, Exclusive breastfeeding, Duration of exclusive breastfeeding, Breastfeeding continuation at 3, 6, and 12 months …

We should note that breastfeeding was defined as the infant receiving breast milk regardless of other liquids or foods. Exclusive breastfeeding was recorded separately as a secondary variable.

The primary outcomes of this study were on breastfeeding continuation at 3, 6, and 12 months postpartum as reported by maternal recall at 12 months postpartum.

### 2.4 Statistical analysis

Data were analyzed using SPSS software (version 26.0, IBM Corp), with Excel used for data presentation and visualization ( https://www.office.com/?omkt=fr-FR ).

Less than 5% of data were missing for any given variable. As missingness was minimal and assumed to be random, analyses were performed using complete-case data without imputation.

Descriptive statistics summarized qualitative variables as frequencies and percentages. Quantitative variables were described using means ± standard deviations (for normally distributed data) or medians with interquartile ranges (for non-normal data). Normality was assessed using skewness, kurtosis, and formal tests such as the Shapiro-Wilk test. ^12^

Analytical methods included the Chi-squared test or Fisher’s exact test for categorical variables, and the Student’s t-test or Mann-Whitney U test for continuous variables depending on distribution. Statistical significance was set at p ≤ 0.05. ^13^

Multivariate logistic regression models were then constructed to identify independent predictors of breastfeeding duration across three categories: 0-3 months, 3-6 months and 6-12 months. Variables with a p value ≤ 0.20 in the univariate analysis were included in the model. Adjusted odds ratios (ORs) and 95% confidence intervals (CIs) were reported. A p value ≤ 0.05 was considered statistically significant. ^14, 15^

## 3. Results

In total, 400 women were included.

### 3.1 Descriptive study

**1. Demographic characteristics**

Most of the participants were Tunisian (396 women, 99.0%), while 4 women (1.0%) were of Sub-Saharan African origin. Regarding residence, 366 women (91.5%) lived in urban areas and 34 (8.5%) in rural areas. A higher education level was reported by 139 women (34.8%), whereas 261 (65.3%) did not have higher education. A low socioeconomic status was observed in 63 women (15.8%), while 337 (84.3%) had a higher socioeconomic level.

The study population predominantly comprised non-working women (65.5%), with blue-collar workers representing 20.3% and white-collar professionals 14.3% of participants ( Table 1).

**Table 1.** Sociodemographic characteristics of the study population.

Demographic characteristic	Category	N	%
**Ethnicity**	**Tunisian**	396	99.0
	**Sub-Saharan African**	4	1.0
**Residence**	**Rural**	34	8.5
	**Urban**	366	91.5
**Higher Education**	**No**	261	65.3
	**Yes**	139	34.8
**Socioeconomic Level**	**Low**	63	15.8
	**Not Low**	337	84.3
**Occupational Status**	**Non-Working**	262	65.5
	**Blue-Collar**	81	20.3
	**White-Collar**	57	14.3

**2. Health and lifestyle factors**

Table 2 represents health and lifestyle factors among the study population.

**Table 2.** Health and lifestyle factors among the study population.

Health & Lifestyle factors	Category	N	%
**Active Smoking**	**No smoking**	394	98.5
	**Smoking**	6	1.5
**Passive Smoking**	**No**	291	72.8
	**Yes**	109	27.3
**Tobacco Exposure**	**No exposure**	296	74.0
	**Active/passive exposure**	104	26.0
**Obesity**	**No obesity**	318	79.5
	**Obesity**	82	20.5
**Surgical History**	**No**	394	98.5
	**Appendectomy**	4	1.0
	**Cholecystectomy**	2	0.5

Active smoking was reported by 6 women (1.5%), while 394 (98.5%) did not smoke. Passive smoking exposure was reported by 109 women (27.3%), and 291 (72.8%) had no exposure. Overall tobacco exposure, whether active or passive, was reported by 104 women (26.0%), while 296 (74.0%) had no exposure.

Obesity was present in 82 women (20.5%), while 318 (79.5%) were not obese.

Regarding surgical history, 6 women (1.5%) had undergone surgery, with 4 (1.0%) having had an appendectomy and 2 (0.5%) a cholecystectomy. The remaining 394 women (98.5%) had no surgical history.

**3. Pregnancy and delivery details**

Pregnancy and delivery details among the study population are summarized in Table 3.

**Table 3.** Pregnancy and delivery details among the study population.

Pregnancy & Delivery details	Category	N	%
**Parity**	**Low parity (Pauciparous)**	211	52.8
	**Multiparous**	189	47.3
**Singleton/Multiple**	**Singleton**	396	99.0
	**Multiple**	4	1.0
**Delivery Mode**	**Cesarean section**	211	52.8
	**Vaginal delivery**	189	47.3
**Encouraged to Move During Labor**	**No**	34	8.5
	**Yes**	366	91.5
**Degree of Urgency**	**Urgent**	108	51.2
	**Non-urgent ("Cold")**	103	48.8
**Delivery Term (Weeks)**	**<37**	34	8.5
	**37-39**	234	58.5
	**39-40**	82	20.5
	**≥40**	50	12.5
**Delivery Term**	**Preterm**	34	8.5
	**Term**	366	91.5
**Macrosomia**	**No**	370	92.5
	**Yes**	30	7.5

Low parity (pauciparous) was reported in 211 women (52.8%), while 189 (47.3%) were multiparous.

A singleton pregnancy was reported in 396 women (99.0%) and a multiple pregnancy in 4 women (1.0%).

Cesarean section was the mode of delivery for 211 women (52.8%), while 189 (47.3%) had a vaginal delivery (AVB). During labor, 366 women (91.5%) were encouraged to move, whereas 34 (8.5%) were not.

Delivery was urgent in 108 cases (51.2%) and non-urgent (“cold”) in 103 cases (48.8%).

Regarding gestational age at delivery, 34 women (8.5%) gave birth before 37 weeks, 234 (58.5%) between 37 and 39 weeks, 82 (20.5%) between 39 and 40 weeks, and 50 (12.5%) at 40 weeks or more. In total, 34 deliveries (8.5%) were preterm, and 366 (91.5%) were term.

Macrosomia was present in 30 newborns (7.5%), while 370 (92.5%) did not have macrosomia.

**4. Postpartum and neonatal outcomes**

Table 4 represents postpartum and neonatal outcomes among the study population.

**Table 4.** Postpartum and neonatal outcomes details among the study population.

Postpartum & Neonatal outcomes	Category	N	%
**Neonatal Unit Admission**	**No**	320	80.0
	**Yes**	80	20.0
**Severe Postpartum Pain**	**No**	214	53.5
	**Yes**	186	46.5
**Skin-to-Skin Contact**	**No**	247	61.8
	**Yes**	153	38.3
**Mother-Child Separation**	**No**	327	81.8
	**Yes**	73	18.3

Admission to a neonatal unit was necessary for 80 newborns (20.0%), while 320 (80.0%) did not require it.

Severe postpartum pain was reported by 186 women (46.5%), while 214 (53.5%) did not report such pain.

Skin-to-skin contact was practiced by 153 women (38.3%), while 247 (61.8%) did not have this contact.

Mother-child separation occurred in 73 cases (18.3%), while 327 women (81.8%) remained with their newborns.

**5. Breastfeeding practices and support**

Breastfeeding practices and support among the study population are summarized in Table 5:

▪ **Breastfeeding experience** was reported by 176 women (44.0%), while 224 (56.0%) did not breastfeed.
▪ **Breastfeeding intention** was reported by 398 women (99.5%), and only 2 (0.5%) had no such intention.
▪ **Breastfeeding education** was received by 184 women (46.0%), while 216 (54.0%) did not receive any.
▪ **The timing of breastfeeding initiation** was as follows:
  - less than 1 hour for 77 women (19.3%),
  - between 1 and 6 hours for 210 (52.5%),
  - between 6 and 12 hours for 66 (16.5%),
  - between 12 and 24 hours for 5 (1.3%),
  - and 24 hours or more for 30 women (7.5%).
  - Twelve women (3.0%) did not initiate breastfeeding at all during hospitalisation.

**Table 5.** Breastfeeding practices and support among the study population.

Breastfeeding practices & Support	Category	N	%
**Breastfeeding Experience**	**No**	224	56.0
	**Yes**	176	44.0
**Intention to Breastfeed**	**No**	2	0.5
	**Yes**	398	99.5
**Breastfeeding Education**	**No**	216	54.0
	**Yes**	184	46.0
**Breastfeeding Initiation Delay**	**<1 hour**	77	19.3
	**1-6 hours**	210	52.5
	**6-12 hours**	66	16.5
	**12-24 hours**	5	1.3
	**≥24 hours**	30	7.5
	**No initiation during hospitalisation**	12	3.0
**Breastfeeding Initiation**	**Delayed**	323	80.8
	**Timely**	77	19.3
**Use of Formula Milk**	**No**	295	73.8
	**Yes**	105	26.3
**Assistance with First Feeding**	**No**	263	65.8
	**Yes**	137	34.3
**Assistance After First Feeding**	**No**	293	73.3
	**Yes**	107	26.8
**Education During Hospitalization**	**No**	293	73.3
	**Yes**	107	26.8
**Family Support**	**No**	110	27.5
	**Yes**	290	72.5

Overall, delayed initiation of breastfeeding was reported in 323 cases (80.8%), while timely initiation was noted in 77 cases (19.3%).

▪ **The use of formula milk** was reported by 105 women (26.3%), while 295 (73.8%) did not use it.
▪ **Assistance with the first feeding** was received by 137 women (34.3%), while 263 (65.8%) did not receive help. After the first feeding, further assistance was reported by 107 women (26.8%), while 293 (73.3%) did not receive additional help.
▪ **Breastfeeding education during hospitalization**: 107 women (26.8%) received breastfeeding education, while 293 (73.3%) did not.
▪ **Family support** was reported by 290 women (72.5%), while 110 (27.5%) did not receive support.

## 4. Analytical study

### 4.1 Univariate analysis

**1. Demographic and socioeconomic factors**

Table 6 represents the univariate analysis of demographic and socioeconomic factors across postpartum breastfeeding duration groups (1–3 months, 3–6 months, and >6 months).

**Table 6.** Univariate analysis of demographic and socioeconomic factors across postpartum breastfeeding duration groups (1–3 months, 3–6 months, and >6 months).

Demographic & Socioeconomic factors	Category	1–3 months N (%)	3–6 months N (%)	>6 months N (%)	*p* -value
**Ethnicity**	**Tunisian**	53 (100%)	104 (96.3%)	239 (100%)	0.064
	**Sub-Saharan African**	0 (0%)	4 (3.7%)	0 (0%)	
**Residence**	**Rural**	4 (7.5%)	11 (10.2%)	19 (7.9%)	0.740
	**Urban**	49 (92.5%)	97 (89.8%)	220 (92.1%)	
**Higher Education**	**No**	38 (71.7%)	69 (63.9%)	154 (64.4%)	0.525
	**Yes**	15 (28.3%)	39 (36.1%)	85 (35.6%)	
**Low Socioeconomic Level**	**No**	44 (83.0%)	80 (74.1%)	213 (89.1%)	**0.005**
	**Yes**	9 (17.0%)	28 (25.9%)	26 (10.9%)	

All participants in the 1–3 month and >6 month postpartum groups were Tunisian (100%), whereas in the 3–6 month group, 104 women (96.3%) were Tunisian and 4 women (3.7%) were Sub-Saharan African. This difference in ethnicity was not statistically significant ( *p* = 0.064).

Concerning residence, most women lived in urban areas across all groups: 92.5% in the 1–3 month group, 89.8% in the 3–6 month group, and 92.1% in the >6 month group. Rural residence was reported by 7.5%, 10.2%, and 7.9% of women respectively. These differences were not significant ( *p* = 0.740).

Regarding education, women without higher education represented 71.7% in the 1–3 month group, 63.9% in the 3–6 month group, and 64.4% in the >6 month group. These variations were not statistically significant ( *p* = 0.525).

However, socioeconomic status showed a significant difference between the groups ( *p* = 0.005). A low socioeconomic level was reported in 17.0% of women in the 1–3 month group, 25.9% in the 3–6 month group, and only 10.9% in the >6 month group.

**2. Health and lifestyle factors**

Table 7 represents the univariate analysis of healthand lifestyle factors across the postpartum breastfeeding duration groups.

**Table 7.** Univariate analysis of health and lifestyle factors across the postpartum breastfeeding duration groups (1–3 months, 3–6 months, and >6 months).

Health & Lifestyle factors	Category	1–3 months	3–6 months	>6 months	*p* -value
Active Smoking	No	51 (96.2%)	107 (99.1%)	236 (98.7%)	0.420
	Yes	2 (3.8%)	1 (0.9%)	3 (1.3%)	
Passive Smoking	No	35 (66.0%)	78 (72.2%)	178 (74.5%)	0.266
	Yes	18 (34.0%)	30 (27.8%)	61 (25.5%)	
Obesity	No	39 (73.6%)	80 (74.1%)	199 (83.3%)	**0.026**
	Yes	14 (26.4%)	28 (25.9%)	40 (16.7%)	

Active smoking was uncommon across all groups, with 3.8% of women smoking in the 1–3 month group, 0.9% in the 3–6 month group, and 1.3% in the >6 month group ( *p* = 0.420; not significant). Passive smoking exposure was reported by 34.0%, 27.8%, and 25.5% of women respectively ( *p* = 0.266; not significant).

Obesity showed a significant difference between groups ( *p* = 0.026). It was reported in 26.4% of women in the 1–3 month group, 25.9% in the 3–6 month group, and only 16.7% in the >6 month group.

**3. Pregnancy and delivery factors**

Cesarean section was the most common delivery mode in all groups, reported in 54.7% of the 1–3 month group, 52.8% of the 3–6 month group, and 52.3% of the >6 month group ( *p* = 0.789; not significant).

Preterm delivery (<37 weeks) occurred in 9.4%, 9.3%, and 7.9% of the women, respectively ( *p* = 0.635; not significant).

Macrosomia was similarly distributed among groups (5.7%, 8.3%, and 7.5%; *p* = 0.864; not significant) (See Table 8).

**Table 8.** Univariate analysis of pregnancy and delivery factors across postpartum breastfeeding duration groups (1–3 months, 3–6 months, and >6 months).

Pregnancy & Delivery factors	Category	1–3 months	3–6 months	>6 months	*p* -value
Delivery Mode	**Cesarean section**	29 (54.7%)	57 (52.8%)	125 (52.3%)	0.789
	**Vaginal delivery**	24 (45.3%)	51 (47.2%)	114 (47.7%)	
Term Delivery	**Preterm (<37 weeks)**	5 (9.4%)	10 (9.3%)	19 (7.9%)	0.635
	**Term (≥37 weeks)**	48 (90.6%)	98 (90.7%)	220 (92.1%)	
Macrosomia	**No**	50 (94.3%)	99 (91.7%)	221 (92.5%)	0.864
	**Yes**	3 (5.7%)	9 (8.3%)	18 (7.5%)	

**4. Breastfeeding practices and support**

The univariate analysis of breastfeeding practices and support factors across postpartum breastfeeding duration groups (1–3 months, 3–6 months, and >6 months) is summarized in Table 9:

▪ **Breastfeeding initiation:**
  - **Timely breastfeeding initiation (within 1 hour)** showed a significant difference between groups ( *p* < 0.001). Only 13.2% of women in the 1–3-month group and 2.8% in the 3–6 month group initiated breastfeeding early, compared to 28.0% in the >6-month group.
  - Consequently, **delayed breastfeeding initiation** was more prevalent in the 1–3 month (86.8%) and 3–6-month (97.2%) groups than in the >6-month group (72.0%), which was also statistically significant ( *p* < 0.001).
▪ **The use of formula milk** was reported by 34.0% of women in the 1–3-month group, 22.2% in the 3–6 month group, and 26.4% in the >6-month group ( *p* = 0.750; not significant).
▪ **Skin-to-skin contact** was practiced in 37.7%, 36.1%, and 39.3% of the cases in the respective groups ( *p* = 0.633; not significant).
▪ **Family support** was present for 79.2% of the 1–3 month group, 71.3% of the 3–6 month group, and 71.5% of the >6-month group ( *p* = 0.450; not significant).
▪ **Breastfeeding continuation**

**Table 9.** Univariate analysis of breastfeeding practices and support factors across postpartum breastfeeding duration groups (1–3 months, 3–6 months, and >6 months).

Breastfeeding practices & Support	Category	1–3 months	3–6 months	>6 months	*p* -value
Timely Initiation (<1h)	Yes	7 (13.2%)	3 (2.8%)	67 (28.0%)	**0.000**
Delayed Initiation	Yes	46 (86.8%)	105 (97.2%)	172 (72.0%)	**0.000**
Formula Use	Yes	18 (34.0%)	24 (22.2%)	63 (26.4%)	0.750
Skin-to-Skin Contact	Yes	20 (37.7%)	39 (36.1%)	94 (39.3%)	0.633
Family Support	Yes	42 (79.2%)	77 (71.3%)	171 (71.5%)	0.450

**Figure 2.**
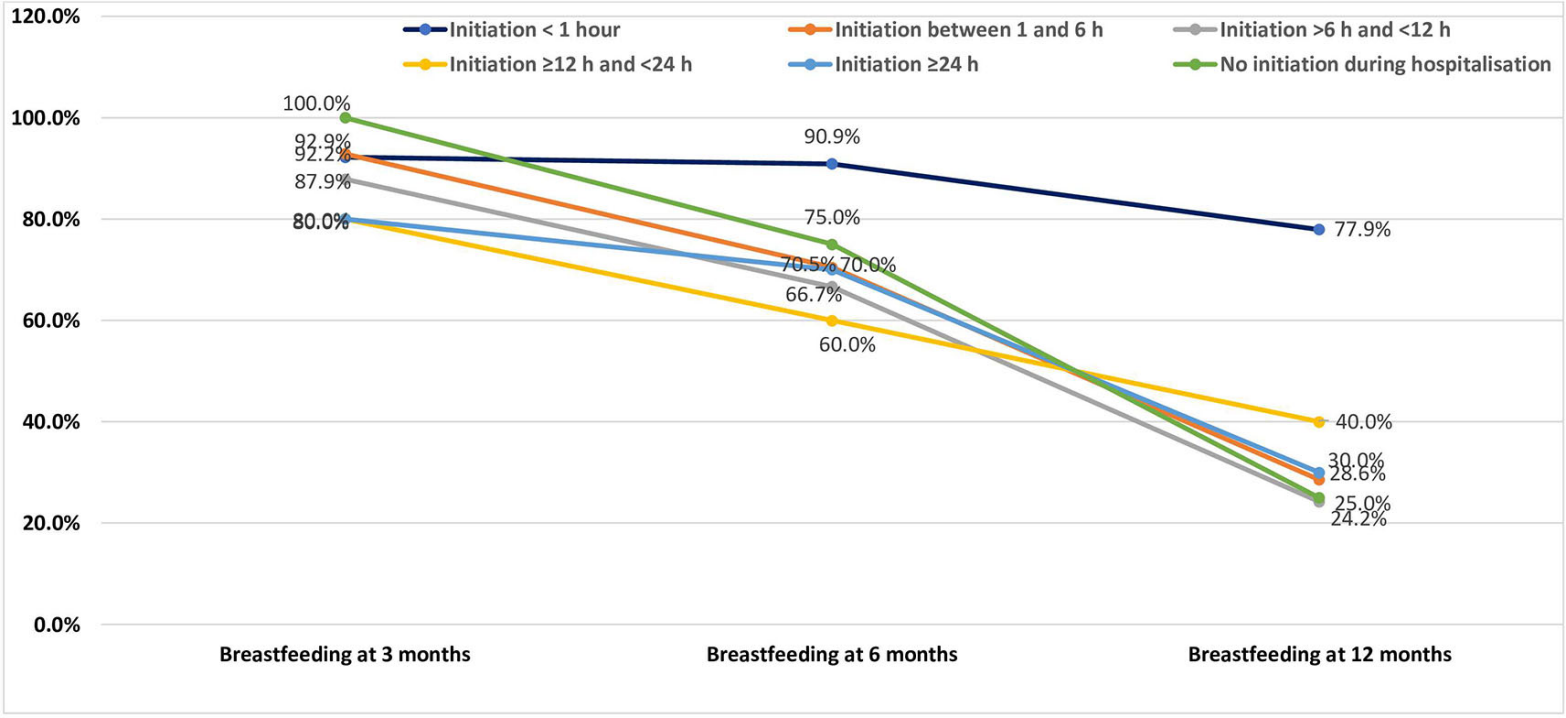
Breastfeeding duration in relation to the timing of breastfeeding initiation among the study population.

An analysis of breastfeeding duration in relation to the timing of breastfeeding initiation reveals a clear trend: the earlier the initiation, the higher the likelihood of sustained breastfeeding, particularly at 6 and 12 months ( Figure 2).

At 3 months postpartum, breastfeeding rates remained relatively high across all initiation groups. Women who initiated breastfeeding within the first hour had a breastfeeding rate of 92.2%, comparable to those who initiated between 1 and 6 hours (92.9%). Even among those who initiated breastfeeding after 24 hours or did not initiate at all during hospitalisation, rates remained high (80.0% and 100.0%, respectively). This suggests that in the short term, other factors—such as initial motivation or support in the early postpartum period—may help maintain breastfeeding regardless of initiation timing.

However, differences became more pronounced at 6 months. Women who initiated breastfeeding within the first hour had the highest rate (90.9%), whereas rates dropped to 70.5% among those who initiated between 1 and 6 hours, and to 66.7% for those initiating between 6 and 12 hours. The lowest rates were seen among those with later initiation (60–70%) and no initiation (75%). These findings suggest that early initiation is linked to better breastfeeding continuity.

At 12 months, the trend was even more marked. The breastfeeding rate remained relatively high in the group that initiated within the first hour (77.9%), while it fell drastically in all other groups. Only 28.6% of women who initiated between 1 and 6 hours, 24.2% of those between 6 and 12 hours, and 25.0% of those who did not initiate at all during hospitalisation were still breastfeeding at 12 months. This demonstrates the long-term benefit of early breastfeeding initiation, especially within the first hour of birth.

### 4.2 Multivariate analysis

Based on the multivariate analysis of factors associated with breastfeeding continuation across different postpartum periods, several key patterns emerge (see Table 10):

▪ **BMI** consistently demonstrated a protective effect throughout all time periods, with each unit increase in BMI associated with approximately 10% lower odds of breastfeeding continuation at 3 months, 12% lower odds at 6 months, and 4% lower odds at 12 months. This consistent relationship suggests that weight management represents an important factor for breastfeeding success across the entire first year postpartum.
▪ **Smoking behavior** showed a time-dependent relationship, with active smoking significantly reducing the odds of breastfeeding continuation by 85% at 3 months and 87% at 6 months, though this effect was no longer statistically significant by 12 months. This pattern indicates that smoking cessation interventions may be particularly crucial for supporting breastfeeding in the early postpartum period.
▪ **Breastfeeding initiation practices** revealed a U-shaped importance, demonstrating strong positive effects at both 3 months (389% increased odds) and 12 months (728% increased odds), while showing no significant association at 6 months. This suggests that early breastfeeding initiation has both immediate and long-lasting benefits for breastfeeding continuation.
▪ **Breastfeeding intention** emerged as an overwhelmingly strong predictor at 3 months, associated with more than 400-fold increased odds of breastfeeding continuation. Though the effect diminished over time, it remained substantial at later time points, highlighting the critical importance of prenatal intention formation for early breastfeeding success.

**Table 10.** Factors associated with exclusive breastfeeding - complete multivariate analysis.

Variable	Breastfeeding continuation at 3 months	Breastfeeding continuation at 6 months	Breastfeeding continuation at 12 months
**BMI**	OR = 0.904 (0.849-0.963)	OR = 0.883 (0.812-0.961)	OR = 0.957 (0.942-0.972)
	*p* = 0.002	*p* = 0.004	*p* < 0.001
**Active Smoking**	OR = 0.155 (0.027-0.879)	OR = 0.133 (0.022-0.792)	OR = 0.245 (0.024-2.538)
	*p* = 0.035	*p* = 0.027	*p* = 0.239
**Breastfeeding Intention**	OR = 428.5 (20.0-9180.1)	OR = 5.107 (0.864-30.195)	OR = 4.398 (0.796-24.296)
	*p* < 0.001	*p* = 0.072	*p* = 0.089
**Breastfeeding Initiation**	OR = 4.892 (2.066-11.588)	OR = 0.993 (0.352-2.800)	OR = 8.278 (4.556-15.040)
	*p* < 0.001	*p* = 0.989	*p* < 0.001
**Low Socioeconomic Status**	OR = 0.859 (0.444-1.658)	OR = 0.768 (0.308-1.911)	OR = 0.496 (0.242-1.015)
	*p* = 0.650	*p* = 0.570	*p* = 0.055
**Ethnicity**	OR = 0.109 (0.011-1.101)	OR = 323.1 (27.9-3737.6)	OR = 0.000 (0.000-.)
	*p* = 0.060	*p* < 0.001	*p* = 0.999
**Skin-to-Skin Contact**	OR = 0.623 (0.368-1.056)	OR = 0.625 (0.283-1.378)	OR = 0.830 (0.485-1.420)
	*p* = 0.079	*p* = 0.244	*p* = 0.496
**Knowledge of Benefits**	OR = 1.068 (0.645-1.768)	OR = 1.353 (0.648-2.825)	OR = 1.619 (0.994-2.638)
	*p* = 0.799	*p* = 0.421	*p* = 0.053
**Higher Education**	OR = 0.943 (0.557-1.597)	OR = 1.775 (0.747-4.218)	OR = 0.991 (0.598-1.642)
	*p* = 0.827	*p* = 0.194	*p* = 0.973
**Feeding Delay (hours)**	OR = 1.001 (0.978-1.024)	OR = 0.976 (0.951-1.002)	OR = 1.017 (0.996-1.039)
	*p* = 0.960	*p* = 0.070	*p* = 0.117

Several factors demonstrated borderline significance that may warrant clinical attention:

▪ **Low socioeconomic status** showed a trend toward reducing breastfeeding odds by approximately 50% at 12 months,
▪ **Knowledge of breastfeeding** benefits approached significance with a 62% increase in odds at 12 months.
▪ **Skin-to-skin contact** demonstrated a borderline protective effect at 3 months, and feeding delay showed a trend toward reducing breastfeeding success at 6 months.

**Higher education** showed no significant association with breastfeeding continuation at any time point, suggesting that educational attainment alone may not determine breastfeeding success.

The inconsistent and extreme values for ethnicity likely reflect statistical instability rather than true biological effects, possibly due to small subgroup sizes.

These findings suggest that breastfeeding support strategies should be tailored to specific postpartum periods, with emphasis on intention formation and smoking cessation prenatally, early initiation practices immediately postpartum, and ongoing socioeconomic support throughout the first year.

## 5. Discussion

The findings from our study examining breastfeeding practices among Tunisian women reveal important similarities and differences when compared with international research.

Our observation of a 44% breastfeeding rate at 12 months postpartum aligns closely with Yalçin et al.’s findings ^16^ in Turkey (55.9%), yet surpasses rates reported in high-income countries like Australia (31.8%) ^17^ and Korea (33.7%). ^18^ This suggests cultural factors in Tunisia may be more supportive of prolonged breastfeeding than in some Western contexts, though substantial room for improvement remains.

A critical finding from our study was the powerful association between early initiation and breastfeeding duration - while only 19.3% of mothers initiated breastfeeding within the first hour, this group accounted for 77.9% of those still breastfeeding at 12 months. This dose-response relationship echoes Meedya’s intervention research ^19^ showing early practices significantly influence long-term outcomes. Our multivariate analysis further quantified these effects: early initiation demonstrated a U-shaped importance, with strong positive effects at 3 months (389% increased odds) and 12 months (728% increased odds), while showing no significant association at 6 months. This indicates that early initiation has both immediate and long-lasting benefits for breastfeeding continuation.

However, our 80.8% delayed initiation rate indicates systemic challenges in immediate postpartum care that mirror Bond et al.’s identification of hospital routines as barriers. ^20^

The analysis of breastfeeding intention highlighted its overwhelming predictive value at 3 months, with more than 400-fold increased odds of continued breastfeeding. Although the effect diminished over time, it remained substantial at later postpartum periods, emphasizing the critical role of prenatal intention formation in early breastfeeding success.

Our study identified maternel obesity (present in 20.5% of participants) as a significant predictor of shorter breastfeeding duration, consistent with the findings of Achike and Akpinar-Elci’s systematic review, ^21^ which analyzed 23 studies on the impact of pregestational maternal BMI on breastfeeding outcomes. Their review showed that elevated maternal BMI prior to pregnancy is linked to reduced intention to breastfeed, delayed initiation, and shorter duration of both exclusive and overall breastfeeding. This association likely reflects a combination of physiological barriers (delayed lactogenesis, hormonal dysregulation), psychological challenges (low confidence, negative body image), and systemic factors (less support, provider bias). ^21^ In our multivariate analysis, maternel BMI also emerged as a consistent determinant across all postpartum periods, with each unit increase in BMI associated with approximately 10% lower odds of breastfeeding continuation at 3 months, 12% lower odds at 6 months, and 4% lower odds at 12 months. This findings underscore the importance of weight management and related support throughout the first postpartum year to optimize breastfeeding outcomes.

Similarly, smoking behavior showed a time-dependent negative effect, with active smoking significantly reducing the odds of breastfeeding continuation by 85% at 3 months and 87% at 6 months, although this association was no longer significant by 12 months. These results suggest that smoking cessation interventions may be particularly crucial in the early postpartum period.

The socioeconomic gradient we observed confirms Chimoriya et al.’s Sydney-based findings, ^22^ though the mechanisms may differ in our context. Low socioeconomic status showed a trend toward reducing breastfeeding odds by ~50% at 12 months, and knowledge of breastfeeding benefits approached significance with a 62% increased odds at 12 months. In contrast, higher educational attainment showed no significant association at any time point, suggesting that formal education alone may not determine breastfeeding success.

Contrary to expectations from Bond et al.’s work, ^20^ we found no association between cesarean delivery (52.8% rate) and breastfeeding outcomes, suggesting cultural or healthcare adaptations may mitigate this typically negative relationship.

Our study was conducted in a tertiary-level maternity hospital (level 3), which manages a high volume of high-risk deliveries, including cesarean sections, preterm births, and neonates requiring intensive care. This setting contributes to institutional barriers to early initiation and skin-to-skin contact: routine postnatal monitoring, separation of mother and infant for neonatal intensive care, and the high workload of healthcare staff can delay the first breastfeeding session. Such organizational constraints are consistent with Bond et al.’s findings on hospital routines as impediments to early breastfeeding. ^20^

In addition, cultural factors in the Tunisian context, such as family influence on postpartum practices and traditional beliefs regarding colostrum and neonatal care, may further delay initiation. Mothers may defer breastfeeding or skin-to-skin contact due to guidance from elders or perceptions that immediate feeding is not essential, reflecting deeply rooted postpartum customs. Together, the tertiary hospital setting and local cultural norms help explain the low rates of early initiation (19.3%) and skin-to-skin contact (38.3%) observed in our cohort.

Postpartum practices documented in our study highlight several evidence-practice gaps. Our low skin-to-skin contact rate falls far below standards advocated by the American Academy of Pediatrics, ^23^ while limited breastfeeding education (46%) contrasts with Park and Ryu’s ^24^ meta-analysis showing education’s effectiveness. Skin-to-skin contact demonstrated a borderline protective effect at 3 months, and delayed feeding showed a trend toward reducing breastfeeding success at 6 months. These implementation shortcomings help explain our population’s challenges, particularly when combined with Niu et al.’s findings ^25^ about the importance of early metabolic programming through breastfeeding.

Our health outcome data contribute new perspectives to existing research. While Delgado et al. ^26^ focused on long-term mental health outcomes and Niu et al. ^25^ examined metabolic benefits, our documentation of immediate postpartum challenges (20% neonatal intensive care unit admission, 46.5% severe pain) identifies understudied barriers to breastfeeding establishment. These findings suggest Cordell and Elverson’s ^27^ call for improved postpartum support applies particularly strongly in our context.

The policy implications emerging from our study both confirm and extend existing recommendations. Our results strongly support implementing the World Health Organisation (WHO)’s “Ten Steps” as analyzed by Binns and Lee, ^28^ while also suggesting adaptations for local factors such as obesity prevalence. Addressing both institutional and cultural barriers, along with reinforcing education and support for mothers, could help improve early initiation, skin-to-skin contact, and overall breastfeeding outcomes in Tunisian tertiary care settings.

Beyond the determinants analyzed in our study, several additional factors recently highlighted in the literature may also influence breastfeeding outcomes. Pham et al. ^29^ reported that maternal burnout, particularly among healthcare professionals, can negatively affect lactation performance and breastfeeding continuation. Their findings emphasize the need to consider maternal psychological well-being and stress management as integral components of breastfeeding promotion strategies, especially in high-pressure hospital environments.

Furthermore, Nguyen et al. ^30^ demonstrated that preterm birth—often resulting from high-risk pregnancies and complex obstetric conditions—has substantial implications for neonatal outcomes and early mother-infant interactions. In our tertiary-level setting, where high-risk and preterm deliveries are frequent, such factors may partly explain the challenges observed in early breastfeeding initiation and skin-to-skin contact.

In addition, Nguyen et al. ^31^ highlighted the role of non-pharmacological interventions, including dietary counseling and physical activity, in the management of gestational diabetes mellitus (GDM). Since GDM and maternal metabolic health directly influence both lactation physiology and postpartum recovery, integrating nutritional and lifestyle support into perinatal care could further enhance breastfeeding success.

Together, these complementary perspectives—addressing psychological stress, high-risk pregnancy, and maternal metabolic health—broaden the understanding of breastfeeding determinants and underscore the need for multidimensional, context-specific interventions to optimize breastfeeding outcomes in tertiary maternity settings such as ours.

### 5.1 Study strengths and limitations

Our study of breastfeeding practices among Tunisian women makes several important contributions to the literature while acknowledging key methodological limitations.

Among its principal strengths, this study provides rare quantitative data from a North African context, addressing a major geographic gap in global breastfeeding research traditionally dominated by Western and Asian datasets. ^32^ By documenting both the very high breastfeeding intention (99.5%) and the substantial decline in actual practice (44% at 12 months), our work highlights the persistent “intention–practice gap,” a phenomenon widely recognized globally but rarely examined in our region.

From an analytical standpoint, the multivariate modeling allowed identification of several robust predictors of breastfeeding continuation:

• Maternal BMI emerged as a consistent determinant across all time periods: each unit increase in BMI was associated with approximately 10% lower odds of breastfeeding continuation at 3 months, 12% lower odds at 6 months, and 4% lower odds at 12 months, confirming obesity as an independent risk factor for early cessation.
• Smoking behavior demonstrated a strong time-dependent effect, reducing breastfeeding odds by 85% at 3 months and 87% at 6 months, underscoring the need for smoking-cessation support early in the postpartum period.
• Conversely, early initiation of breastfeeding showed a “U-shaped” importance, exerting marked positive effects both at 3 months (aOR ≈ 4.9) and 12 months (aOR ≈ 8.3), while breastfeeding intention emerged as an exceptionally powerful predictor in early postpartum (aOR ≈ 400).

These findings collectively emphasize that breastfeeding outcomes in our context are driven by modifiable clinical and behavioral factors, rather than immutable demographic characteristics. The documentation of 46.5% severe postpartum pain and low skin-to-skin contact (38.3%) further identifies actionable targets for improving perinatal care quality.

However, several limitations must be carefully considered:

• The study was conducted in a single tertiary-level maternity hospital in the capital, serving a predominantly urban (91.5%) and high-socioeconomic (84.3%) population. As such, the results are not representative of rural or lower-income Tunisian women, who likely face distinct sociocultural and healthcare barriers. This single-site, high-socioeconomic sample limits the generalizability of our findings to the broader national population.
• Furthermore, breastfeeding duration and related behaviors were self-reported retrospectively, which introduces potential recall and social desirability bias. The absence of direct observation or longitudinal follow-up precludes validation of reported practices. Unmeasured confounders such as maternal employment, parity, and Baby-Friendly Hospital Initiative (BFHI) certification may also have influenced outcomes.
• Finally, as a cross-sectional analysis, the study cannot infer causality between identified determinants and breastfeeding duration. The lack of data on exclusive versus partial breastfeeding further limits comparability with WHO recommendations.

Despite these constraints, the study’s detailed temporal categorization of breastfeeding initiation and its context-specific findings provide valuable evidence for clinical and policy action in Tunisia and comparable middle-income settings.

### 5.2 Implications

Our findings underscore a pronounced discrepancy between maternal intention and practice, pointing to several modifiable, time-sensitive determinants that can be targeted through policy and clinical interventions:

1. **Prioritize early initiation of breastfeeding “Golden Hour”:** Immediate and uninterrupted skin-to-skin contact after birth should be institutionalized, including during cesarean deliveries when medically feasible.
2. **Institutionalize skin-to-skin contact as a quality metric:** Routine staff training, adherence audits and inclusion of breastfeeding indicators in hospital performance evaluations are essential for aligning with WHO-BFHI standards. ^33, 34^
3. **Address modifiable biological barriers:** Maternal obesity, identified as a consistent predictor of shorter breastfeeding duration warrants targeted antenatal counseling and postnatal lactation support involving obstetricians, midwives and nutritionists.
4. **Optimize postpartum pain management:** The high prevalence of severe pain (46.5%) suggests an overlooked impediment to breastfeeding; multimodal pain-relief strategies compatible with lactation should be standardized.
5. **Promote smoking-cessation interventions:** The strong negative association between smoking and early breastfeeding continuation highlights the importance of integrating cessation counseling into prenatal and postpartum care.
6. **Enhance breastfeeding education and family engagement:** Low education exposure (46%) and limited partner or family involvement emphasize the need for community-based programs that address cultural beliefs and encourage supportive postpartum environments.
7. **Leverage recent labor-policy advances**: Implementing workplace lactation rooms, flexible schedules and awareness campaigns would sustain breastfeeding among working mothers beyond hospital discharge.

Collectively, these strategies would help bridge the gap between maternal motivation and sustained breastfeeding practice in Tunisia.

### 5.3 Recommendations for further research

Future investigations should address the limitations of the present study and expand its scope through:

1. Multicenter longitudinal cohort studies covering both urban and rural populations to assess generalizability and track breastfeeding continuation beyond 12 months.
2. Intervention trials evaluating the impact of structured hospital programs such as early initiation protocols, pain management and tailored lactation counselling on short- and long-term breastfeeding outcomes.
3. Qualitative research exploring sociocultural perceptions, gender norms and healthcare provider attitudes influencing breastfeeding behaviors in Tunisian and North African contexts.
4. Economic evaluations quantifying the cost-effectiveness of implementing BFHI standards and workplace lactation support at scale.
5. Digital and AI-assisted support innovations, assessing their feasibility and acceptability for postpartum women with limited healthcare access.
6. Research on maternal mental health and burnout, as emerging evidence links psychological distress with suboptimal breastfeeding, underscoring the need for integrative maternal care.

## 6. Conclusions

This study provides robust, context-specific evidence that early initiation of breastfeeding within the first hour of life is a decisive factor for sustained breastfeeding up to 12 months in Tunisia. Despite high maternal motivation, systemic barriers including delayed initiation, limited skin-to-skin contact, postpartum pain, maternal obesity and smoking continue to hinder breastfeeding success.

Strengthening early postpartum hospital practices, integrating mental-health and pain-management support, and promoting culturally adapted education are key to improving outcomes. Aligning Tunisian maternity services with WHO “Ten Steps” and BFHI principles, while addressing local constraints, could significantly enhance maternal-child health and contribute to achieving global breastfeeding targets.

## Ethical considerations

Ethical approval for this study was obtained from the Institutional Ethics Committee of Charles Nicolle Hospital, Tunis, Tunisia (Approval number: FWA00032748 – IORG0011243) on October 24, 2023, prior to data collection.

## Consent to participate

Verbal informed consent was selected instead of written consent due to the specific context of the study population. Data were collected from postpartum women during the immediate or early postnatal period, when participants were often physically exhausted, emotionally vulnerable, and had limited time. Requesting written consent in this setting risked creating additional stress or discouraging participation, particularly among women with lower literacy levels.

This approach was reviewed and approved by the Institutional Ethics Committee of Charles Nicolle Hospital as the study involved minimal risk, collected no identifying or sensitive information, and ensured complete anonymity of responses.

All participants received clear information about the study objectives and procedures. Verbal informed consent was obtained, emphasizing the voluntary nature of participation and the right to withdraw at any time without consequence. Confidentiality was strictly maintained: no identifying information was collected, and all responses were recorded anonymously.

## Data Availability

All data sets can be assessed and all study findings reported in the article are shared via Harvard. Harvard Dataverse: “Factors Associated with Breastfeeding Duration in Tunisia: A Cross-Sectional Analysis Highlighting the Role of Early Initiation”, https://doi.org/10.7910/DVN/PXCDRZ. ^35^ This project contains the following:
• Dataset Breastfeeding final English • Breastfeeding findings Harvard Dataverse: “Factors Associated with Breastfeeding Duration in Tunisia: A Cross-Sectional Analysis Highlighting the Role of Early Initiation”, https://doi.org/10.7910/DVN/PXCDRZ. ^35^ This project contains the following:
• Breastfeeding Questionnaire. Data are available under the terms of the Creative Commons Zero “No rights reserved” data waiver (CC0 1.0 Public domain dedication).
