# Supplementary material for: Factors Associated with Breastfeeding Duration in Tunisia: A Cross-Sectional Analysis Highlighting the Role of Early Initiation
Source: F1000Res. 2025 Dec 12;14:881. Originally published 2025 Sep 9. [Version 2] doi: 10.12688/f1000research.169267.2 (PMC12809030; doi:10.12688/f1000research.169267.2)
Supplement: Peer review history

# Reviewer report: Reviewer response for version 2

**DOI:** 10.5256/f1000research.190845.r440947

**Published:** 2026-01-15

**Authors:** Phuc Nhon Nguyen

**Affiliations:** 1 Tu Du Hospital, Ho Chi Minh City, Vietnam

Thank you for your responses and your revision. The paper is well-improved.

Is the work clearly and accurately presented and does it cite the current literature?

Partly

If applicable, is the statistical analysis and its interpretation appropriate?

Partly

Are all the source data underlying the results available to ensure full reproducibility?

Partly

Is the study design appropriate and is the work technically sound?

Partly

Are the conclusions drawn adequately supported by the results?

Partly

Are sufficient details of methods and analysis provided to allow replication by others?

Partly

Reviewer Expertise:

NA

I confirm that I have read this submission and believe that I have an appropriate level of expertise to confirm that it is of an acceptable scientific standard.

# Reviewer report: Reviewer response for version 2

**DOI:** 10.5256/f1000research.190845.r440948

**Published:** 2025-12-30

**Authors:** Samina Naeem Khalid

**Affiliations:** 1 MNCH (Maternal, Neonatal and Child Health) Department, Health Services Academy, Islamabad, ICT, Pakistan

I have no further suggestions. Required changes have been incorporated.

Is the work clearly and accurately presented and does it cite the current literature?

Yes

If applicable, is the statistical analysis and its interpretation appropriate?

Yes

Are all the source data underlying the results available to ensure full reproducibility?

Yes

Is the study design appropriate and is the work technically sound?

Partly

Are the conclusions drawn adequately supported by the results?

Yes

Are sufficient details of methods and analysis provided to allow replication by others?

Partly

Reviewer Expertise:

Maternal and Child Health, Family Planning, Nutrition, Adolescent Health

I confirm that I have read this submission and believe that I have an appropriate level of expertise to confirm that it is of an acceptable scientific standard.

# Reviewer report: Reviewer response for version 1

**DOI:** 10.5256/f1000research.186563.r425108

**Published:** 2025-11-10

**Authors:** Phuc Nhon Nguyen

**Affiliations:** 1 Tu Du Hospital, Ho Chi Minh City, Vietnam

In introduction, maternal milk bank should be also mentioned.

 In table 1, career/ occupation should be added.

 Concomitant diseases should be also added.

 Complications relating to breast among selected population should be described.

 Univariate and multivariate logistic regression should be added to reveal the factors relating to breastfeeding practice greater and less than 6 months.

 Burnout may be a factor influencing to breastfeeding practice. Please discuss. (Pham, et al., 2025) - Ref 1

 Preterm birth in high-risk pregnancy should be discussed. (Nguyen, et al., 2024) - Ref 2

 Diet and physical activity in managing gestational diabetes should be discussed. (Nguyen, et al., 2024) - Ref 3

 English editing is required. For example: Interventions promoting early

Is the work clearly and accurately presented and does it cite the current literature?

Partly

If applicable, is the statistical analysis and its interpretation appropriate?

Partly

Are all the source data underlying the results available to ensure full reproducibility?

Partly

Is the study design appropriate and is the work technically sound?

Partly

Are the conclusions drawn adequately supported by the results?

Partly

Are sufficient details of methods and analysis provided to allow replication by others?

Partly

Reviewer Expertise:

Clinical management in high-risk pregnancies.

I confirm that I have read this submission and believe that I have an appropriate level of expertise to confirm that it is of an acceptable scientific standard, however I have significant reservations, as outlined above.

## References

[1] : Burnout Syndrome and Sexual Disorders Among Vietnamese Female Nurses and Midwives at Tu Du Hospital: A Frontline Hospital-Based Cross-Sectional Study. *Women's Health Reports* .2025;6(1) : 10.1089/whr.2024.0193 660-670 10.1089/whr.2024.0193 PMC1224184440642180

[2] : Neonatal outcomes in the surgical management of placenta accreta spectrum disorders: a retrospective single-center observational study from 468 Vietnamese pregnancies beyond 28 weeks of gestation. *BMC Pregnancy and Childbirth* .2024;24(1) : 10.1186/s12884-024-06349-7 10.1186/s12884-024-06349-7 38566074 PMC10986094

[3] : Non-Pharmacological Management of Gestational Diabetes Mellitus with a High Fasting Glycemic Parameter: A Hospital-Based Study in Vietnam. *Journal of Clinical Medicine* .2024;13(19) : 10.3390/jcm13195895 10.3390/jcm13195895 PMC1147815339407955

# Response

**Published:** 2025-11-12

**Authors:** Narjes karmous

**Affiliations:** University of Tunis El Manar Faculty of Medicine of Tunis, Tunis, Tunis, Tunisia

Dear Dr Nguyen,

 We sincerely thank you for your careful reading of our manuscript and for the constructive and insightful comments which have greatly helped us improve the quality and clarity of the study.

**Reviewer Comment 1: In the Introduction section, maternal milk bank should also be mentioned.**

**Response:**

 The Introduction has been revised to include a brief discussion of maternal milk banks and their role in supporting breastfeeding in high-risk populations, highlighting their relevance for infants unable to receive direct maternal breastfeeding.

**Reviewer Comment 2:  Career/occupation should be added in Table 1.**

**Response:**

 Table 1 has been updated to include maternal occupation/career, providing additional socio-demographic context of the study population.

**Reviewer Comment 3 – Statistical analysis: univariate and multivariate logistic regression should be added for breastfeeding ≥6 months vs. <6 months.**

**Response:**

 We thank you Dr Nguyen for this valuable suggestion. We conducted univariate and multivariate logistic regression analyses to identify independent predictors of breastfeeding duration across three categories: 0-3 months, 3-6 months and 6-12 months. Results are now presented in a new Table (Table 10) and described in the Results section.

**Reviewer Comment 4 – Burnout may influence breastfeeding; please discuss (Pham et al., 2025).**

**Response:**

 We now discuss the potential role of maternal burnout, referencing Pham et al., 2025, in the Discussion section. While our study did not directly measure burnout, evidence suggests it may influence maternal capacity to maintain breastfeeding, particularly in demanding work environments.

**Reviewer Comment 5 – Preterm birth in high-risk pregnancy should be discussed (Nguyen et al., 2024).**

**Response:**

 The Discussion now briefly considers preterm birth as a factor affecting breastfeeding duration, citing Nguyen et al., 2024. We highlight that preterm infants may face additional challenges in establishing breastfeeding, and maternal support strategies are critical.

**Reviewer Comment 6 – Diet and physical activity in gestational diabetes management should be discussed (Nguyen et al., 2024, Ref 2).**

**Response:**

 A paragraph has been added discussing non-pharmacological management of gestational diabetes, including diet and physical activity, and their potential impact on lactation outcomes, referencing Nguyen et al., 2024.

**Reviewer Comment 7 – English editing is required. Example: “Interventions promoting early …”**

**Response:**

 The manuscript has undergone careful professional English editing to improve clarity, grammar, and scientific readability. All sentences have been reviewed to ensure correct syntax and terminology.

**References Updated:**

1. Pham TH, Vo MT, Nguyen PN. Burnout Syndrome and Sexual Disorders Among Vietnamese Female Nurses and Midwives at Tu Du Hospital: A Frontline Hospital-Based Cross-Sectional Study. Women’s Health Rep (New Rochelle). 2025;6(1):660-670.
2. Nguyen PN, Vuong ADB, Pham XTT. Neonatal outcomes in the surgical management of placenta accreta spectrum disorders: a retrospective single-center observational study from 468 Vietnamese pregnancies beyond 28 weeks of gestation. BMC Pregnancy Childbirth. 2024 Apr 2;24(1):228.
3. Nguyen HG, Nguyen KTH, Nguyen PN. Non-Pharmacological Management of Gestational Diabetes Mellitus with a High Fasting Glycemic Parameter: A Hospital-Based Study in Vietnam. J Clin Med. 2024 Oct 2;13(19):5895.

# Reviewer report: Reviewer response for version 1

**DOI:** 10.5256/f1000research.186563.r426656

**Published:** 2025-11-03

**Authors:** Samina Naeem Khalid

**Affiliations:** 1 MNCH (Maternal, Neonatal and Child Health) Department, Health Services Academy, Islamabad, ICT, Pakistan

**Peer Review Report**

**Manuscript:** "Beyond Intentions: The Breastfeeding Intention-Practice Gap in Tunisia- A Cross-Sectional Analysis of Initiation and Continuation among Post-Partum Women" [version 1]

**General Comments**

 The authors present a timely and relevant cross-sectional study examining the critical gap between breastfeeding intention and practice in Tunisia. The topic is of significant public health importance, and the focus on early initiation and modifiable factors is a strength. The manuscript is generally well-structured, and the statistical analysis appears sound. The finding that early initiation within the first hour is a powerful predictor of continuation at 12 months is a compelling and actionable result.

 However, the manuscript in its current form has some limitations that need to be addressed. The most significant issues are the mismatch between the study's actual design and its framing (particularly in the introduction and title), a lack of clarity in the methodology, and an overinterpretation of the temporal relationship between variables due to the cross-sectional design. Addressing these points will significantly strengthen the paper and the validity of its conclusions.

**Issues**

**1. Misalignment of Study Design, Title, and Introduction:**

- **Issue:** The title and introduction frame the study around the "Intention-Practice Gap." However, the results do not directly analyze this gap. The study reports a 99.5% intention rate but does not track how many of these women actually initiated or continued breastfeeding, which is the core of an intention-practice analysis. The study is, in practice, an analysis of factors associated with breastfeeding *duration*, with initiation timing being the key factor. The introduction cites literature from 2024 and 2025, which is anachronistic for a study conducted in 2023-2024 and gives a misleading impression of the study's temporal context.
- **Recommendation:**
  - **Title:** Revise the title to more accurately reflect the actual analysis, e.g., "Factors Associated with Breastfeeding Duration in Tunisia: A Cross-Sectional Analysis Highlighting the Critical Role of Early Initiation."
  - **Introduction:** The introduction should focus on the known factors affecting breastfeeding duration in the region, with a specific hypothesis about the role of initiation timing. Remove the futuristic 2025 framing and cite literature contemporary with or preceding the study period.
  - **Analysis:** If the intention-practice gap is a core aim, the analysis must be reframed. The authors could create a new variable (e.g., "Intention Fulfilled" for women who intended to breastfeed AND were still doing so at 12 months vs. "Intention Not Fulfilled") and analyze predictors of this outcome.

**2. Critical Methodological Clarifications:**

- **Issue 1 (Timing of Data Collection):** The methodology states that breastfeeding duration was measured "up to 12 months postpartum" and was based on "maternal recall at 12 months postpartum." This implies that women were recruited and provided data at a single time point, 12 months after delivery. However, the study period is listed as Nov 2023 - Feb 2024, and it includes "women who delivered during the study period." This creates confusion. Were women interviewed at their 12-month postpartum mark, meaning the study included women who had delivered from late 2022 to early 2023? Or was this a prospective study where women were enrolled at delivery and followed for 12 months? This must be explicitly clarified, as it fundamentally affects the study design (retrospective vs. prospective) and the potential for recall bias.
- **Recommendation:** The authors must clearly state the single time point at which data were collected from each participant. A flowchart would be beneficial here. If it was a retrospective recall at 12 months, this must be explicitly stated as a key limitation.
- **Issue 2 (Definition of Breastfeeding):** The term "breastfeeding" is used throughout without specifying whether it refers to *exclusive*, *predominant*, or *any* breastfeeding. This is a major oversight, as the implications for public health and the comparison with other studies (e.g., WHO targets) depend entirely on this definition. Table 5 mentions "Exclusive breastfeeding," but it is not used in the primary analysis.
- **Recommendation:** Clearly define the outcome variable "breastfeeding" in the methods section (e.g., "any breastfeeding," meaning the infant received breast milk regardless of other liquids or foods). The analysis of exclusive breastfeeding should be integrated into the main results or presented as a secondary outcome.

**3. Limitations of Cross-Sectional Design and Causality:**

- **Issue:** The language in the abstract, results, and discussion often implies a causal relationship (e.g., "Early initiation strongly supports sustained breastfeeding," "Obesity predicted shorter duration"). A cross-sectional study can only demonstrate association, not causation or prediction. The direction of the relationship is not always clear; for instance, mothers who are more motivated to breastfeed long-term may also be more likely to insist on early initiation, rather than early initiation itself causing longer duration.
- **Recommendation:** Use the words "predicts," "supports," "impacts" with "is associated with," "is linked to," "correlates with." The discussion should explicitly acknowledge that the temporal sequence and causality cannot be confirmed from this design.

**4. Sample Representativeness and Generalizability:**

- **Issue:** The sample is highly homogenous (99% Tunisian, 91.5% urban, 84.3% high SES) and from a single, tertiary hospital in the capital. This severely limits the generalizability of the findings to the broader Tunisian population, particularly rural and lower-income women who likely face different barriers.
- **Recommendation:** This must be highlighted as a major limitation in the discussion. The authors should explicitly state that the results are representative of an urban, higher-SES population attending a tertiary center in Tunis and may not be generalizable to all of Tunisia.

**5. Conceptual Framing:**

 The term “intention–practice gap” is compelling but could be theoretically grounded in models such as the **Theory of Planned Behavior** or **Health Belief Model** to strengthen interpretive depth.

**6. Generalizability:**

 The urban-heavy, single-hospital sample (91.5% urban, 99% Tunisian) limits external validity. Future studies should aim for a multi-site design including rural and underserved populations.

**Specific Recommendations**

**Section**

**Recommendation**

**Abstract**

 Add a brief mention of the primary outcome (breastfeeding continuation at 12 months) and main statistical associations (early initiation, obesity).

**Methods**

 Clarify whether sampling was consecutive or convenience-based; specify how missing data were treated; indicate use of multivariate models.

**Results**

 Present key p-values and effect estimates in the text for clarity. Include a summary figure showing breastfeeding continuation by initiation timing.

**Discussion**

 Expand on cultural and hospital-level barriers affecting early initiation and skin-to-skin contact. Consider integrating a short subsection on policy implications specific to Tunisia’s maternal health system.

**Limitations**

 Strengthen this section with more explicit statements about recall bias, single-site limitation, and potential self-report inaccuracies.

**References**

 The references are recent and relevant; however, a few WHO/UNICEF regional breastfeeding policy documents (MENA-focused) could be added for contextual depth.

**Minor Points**

- **Abstract:** The abstract mentions "modifiable barriers such as obesity." While obesity is a modifiable condition at a population level, it is not a readily modifiable factor in the immediate postpartum period. This should be rephrased for clarity.
- **Results:**
  - The tables in the main text (1-9) are presented as placeholders with incomplete data (e.g., "Demo… Cate…"). Full, properly formatted tables must be provided.
  - The p-values for socioeconomic status (p=0.005) and obesity (p=0.026) are noted, but effect sizes (e.g., Odds Ratios from a multivariate regression) are missing. A multivariate analysis controlling for potential confounders (e.g., SES, education, obesity, delivery mode) would greatly strengthen the analytical study section and confirm that early initiation is an independent predictor.
- **Discussion:**
  - The discussion is lengthy and sometimes repetitive. It could be streamlined by focusing on the key findings: the powerful association of early initiation, the role of obesity, and the surprising lack of association with c-section and family support.
  - The comparison of the 12-month rate to other countries is interesting, but the discussion would benefit from more comparison with other North African or Middle Eastern studies, given the cultural similarities.
- **References:** Some references are incomplete or contain errors (e.g., Reference 1 is a press release, not a formal report; Reference 5 is unclear). The reference list should be checked for consistency and completeness against standard citation styles.

**Conclusion**

 This study addresses an important topic and uncovers a very strong and clinically relevant association between early breastfeeding initiation and duration in a Tunisian cohort. The authors have collected valuable data. However, the manuscript requires major revisions to accurately represent its design, clarify critical methodological points, and temper its conclusions to align with the cross-sectional evidence. Once these issues are addressed, it has the potential to be a valuable contribution to the literature on breastfeeding practices in North Africa.

Is the work clearly and accurately presented and does it cite the current literature?

Yes

If applicable, is the statistical analysis and its interpretation appropriate?

Yes

Are all the source data underlying the results available to ensure full reproducibility?

Yes

Is the study design appropriate and is the work technically sound?

Partly

Are the conclusions drawn adequately supported by the results?

Yes

Are sufficient details of methods and analysis provided to allow replication by others?

Partly

Reviewer Expertise:

Maternal and Child Health, Family Planning, Nutrition, Adolescent Health

I confirm that I have read this submission and believe that I have an appropriate level of expertise to confirm that it is of an acceptable scientific standard, however I have significant reservations, as outlined above.

# Response

**Published:** 2025-11-12

**Authors:** Narjes karmous

**Affiliations:** University of Tunis El Manar Faculty of Medicine of Tunis, Tunis, Tunis, Tunisia

Dear Dr Khalid,

 We sincerely thank you for your careful reading of our manuscript and for the constructive and insightful comments which have greatly helped us improve the quality and clarity of the study.

**Reviewer comment 1: Conceptual Framing- The term “intention–practice gap” is compelling but could be theoretically grounded in models such as the Theory of Planned Behavior or Health Belief Model to strengthen interpretive depth.**

**Response:**

 Thank you Dr Khalid for this insightful suggestion. We have now explicitly linked the concept of the “intention–practice gap” to the Theory of Planned Behavior in the Introduction. This framework helps interpret how maternal intentions translate into actual breastfeeding practices and provides a stronger theoretical grounding for our analysis.

**Reviewer comment 2: Title- Revise the title to more accurately reflect the actual analysis, e.g., "Factors Associated with Breastfeeding Duration in Tunisia: A Cross-Sectional Analysis Highlighting the Critical Role of Early Initiation."**

**Response:**

 The title has been revised to: **“Factors Associated with Breastfeeding Duration in Tunisia: The Critical Role of Early Initiation”**. This more accurately reflects the study’s focus on breastfeeding duration and the primary exposure of interest (timing of initiation).

**Reviewer Comment 3: Abstract- Add a brief mention of the primary outcome (breastfeeding continuation at 12 months) and main statistical associations (early initiation, obesity). Use the words "predicts," "supports," "impacts" with "is associated with," "is linked to," "correlates with." Rephrase “modifiable barriers such as obesity”.**

**Response:**

 The Abstract has been revised to:

- Explicitly mention the primary outcome: breastfeeding continuation at 12 months.
- Report key associations: early initiation and maternal obesity.
- Replaced causal language with neutral expressions such as “is associated with”.
- Rephrased obesity-related text to clarify population-level modifiability, now stating: “Despite high breastfeeding intention, delays in initiation, obesity and limited breastfeeding education hinder optimal breastfeeding outcomes”.

**Reviewer Comment 4: Introduction- Focus on known factors affecting breastfeeding duration in the region; specify hypothesis about initiation timing.**

**Response:**

 The Introduction has been revised to:

- Emphasize regional evidence on factors influencing breastfeeding duration.
- Clearly state the study hypothesis: “focusing on factors influencing breastfeeding continuation, particularly the timing of initiation.”

**Reviewer Comment 5: The introduction cites literature from 2024 and 2025, which is anachronistic for a study conducted in 2023-2024 and gives a misleading impression of the study's temporal context.** **Remove the futuristic 2025 framing and cite literature contemporary with or preceding the study period.**

**Response: **Thank you, Dr Khalid, for this comment. We have clarified in the Introduction that “The study included women who delivered between November 2023 and February 2024. Breastfeeding continuation was assessed up to 12 months postpartum, with data collection extending into early 2025.” This clarifies that the 2025 references reflect the period of data collection and analysis, not a future study date. Consequently, the references from 2024–2025 are now temporally appropriate and contemporary to the study period.

**Reviewer Comment 6: Methods- Sampling, Missing Data, Multivariate Models:**

**Clarify whether sampling was consecutive or convenience-based, how missing data were treated, and use of multivariate models.**

**Response:**

- Sampling was consecutive of all eligible women delivering at the study hospital during the study period (Nov 2023 – Feb 2024).
- Less than 5% of data were missing for any given variable. As missingness was minimal and assumed to be random, analyses were performed using complete-case data without imputation.
- Multivariate logistic regression models are now clearly described.

**Reviewer Comment 7: Methods – Breastfeeding Measurement / Study Design:**

**Clarify whether data were collected prospectively or retrospectively and specify time points; define breastfeeding type.**

**Response:**

- The study period, defined as the period of participant inclusion, was from November 1, 2023, to February 29, 2024, during which all women delivering at the department were consecutively invited to participate. Baseline sociodemographic, obstetric, and early postpartum breastfeeding data were collected within the immediate postpartum period. Breastfeeding duration up to 12 months postpartum was obtained retrospectively by maternal recall at the 12-month mark. Therefore, the study represents a cross-sectional analysis with retrospective ascertainment of breastfeeding duration.
- We have now added an explicit statement acknowledging the potential for recall bias in the Study strengths and limitations section

- Breastfeeding is now clearly defined as the infant receiving breast milk regardless of other liquids or foods.

**Reviewer Comment 8 – Results- Include key p-values, effect estimates, and a figure for breastfeeding continuation by initiation timing. Avoid causal language.**

**Response:**

- All key p-values and effect estimates (Odds Ratios, 95% CI) have been added in the text and Tables 3–6.
- A figure illustrating breastfeeding continuation by initiation timing is already included in the manuscript (Figure 2), titled “Breastfeeding duration in relation to the timing of breastfeeding initiation among the study population.” This figure presents the distribution of breastfeeding continuation across initiation categories and directly addresses the reviewer’s request.
- Causal terms have been replaced by neutral phrases (“is associated with”, “correlates with”).

**Reviewer Comment 9: Discussion- Focus on key findings, discuss cultural/hospital-level barriers, and include regional comparisons. Avoid repetition. Replace causal language.**

**Response:**

- The Discussion now emphasizes the strong association of early initiation, the role of maternal obesity, and the lack of association with c-section and family support.
- A subsection on cultural and hospital-level barriers and policy implications in Tunisia has been added.
- Comparative discussion now includes North African and Middle Eastern studies.
- Repetitive statements were removed, and causal language replaced with neutral terms.

**Reviewer Comment 10: Limitations / Generalizability: Emphasize recall bias, single-site limitation, sample homogeneity, and representativeness.**

**Response:**

- Limitations now explicitly mention recall bias, single-site data, and self-report inaccuracies.
- Sample characteristics are described in detail; generalizability is limited to urban, higher-SES women attending a tertiary center in Tunis.
- Recommendation for future multi-site studies including rural and underserved populations is added.

**Reviewer Comment 11: References- Check for completeness and include regional WHO/UNICEF breastfeeding policy documents.**

**Response:**

- All references have been checked for completeness and correctness per Vancouver style.
- Additional references on MENA regional breastfeeding policies were added for context.
